# Genome-Wide Analysis of Yeast Metabolic Cycle through Metabolic Network Models Reveals Superiority of Integrated ATAC-seq Data over RNA-seq Data

**DOI:** 10.1128/msystems.01347-21

**Published:** 2022-06-13

**Authors:** Müberra Fatma Cesur, Tunahan Çakır, Pınar Pir

**Affiliations:** a Computational Systems Biology Group, Department of Bioengineering, Gebze Technical University, Gebze, Kocaeli, Turkey; Wageningen University

**Keywords:** RNA-seq, ATAC-seq, genome-scale metabolic network, flux balance analysis, *Saccharomyces cerevisiae*, yeast metabolic cycle

## Abstract

Saccharomyces cerevisiae undergoes robust oscillations to regulate its physiology for adaptation and survival under nutrient-limited conditions. Environmental cues can induce rhythmic metabolic alterations in order to facilitate the coordination of dynamic metabolic behaviors. Of such metabolic processes, the yeast metabolic cycle enables adaptation of the cells to varying nutritional status through oscillations in gene expression and metabolite production levels. In this process, yeast metabolism is altered between diverse cellular states based on changing oxygen consumption levels: quiescent (reductive charging [RC]), growth (oxidative [OX]), and proliferation (reductive building [RB]) phases. We characterized metabolic alterations during the yeast metabolic cycle using a variety of approaches. Gene expression levels are widely used for condition-specific metabolic simulations, whereas the use of epigenetic information in metabolic modeling is still limited despite the clear relationship between epigenetics and metabolism. This prompted us to investigate the contribution of epigenomic information to metabolic predictions for progression of the yeast metabolic cycle. In this regard, we determined altered pathways through the prediction of regulated reactions and corresponding model genes relying on differential chromatin accessibility levels. The predicted metabolic alterations were confirmed via data analysis and literature. We subsequently utilized RNA sequencing (RNA-seq) and assay for transposase-accessible chromatin using sequencing (ATAC-seq) data sets in the contextualization of the yeast model. The use of ATAC-seq data considerably enhanced the predictive capability of the model. To the best of our knowledge, this is the first attempt to use genome-wide chromatin accessibility data in metabolic modeling. The preliminary results showed that epigenomic data sets can pave the way for more accurate metabolic simulations.

**IMPORTANCE** Dynamic chromatin organization mediates the emergence of condition-specific phenotypes in eukaryotic organisms. Saccharomyces cerevisiae can alter its metabolic profile via regulation of genome accessibility and robust transcriptional oscillations under nutrient-limited conditions. Thus, both epigenetic information and transcriptomic information are crucial in the understanding of condition-specific metabolic behavior in this organism. Based on genome-wide alterations in chromatin accessibility and transcription, we investigated the yeast metabolic cycle, which is a remarkable example of coordinated and dynamic yeast behavior. In this regard, we assessed the use of ATAC-seq and RNA-seq data sets in condition-specific metabolic modeling. To our knowledge, this is the first attempt to use chromatin accessibility data in the reconstruction of context-specific metabolic models, despite the extensive use of transcriptomic data. As a result of comparative analyses, we propose that the incorporation of epigenetic information is a promising approach in the accurate prediction of metabolic dynamics.

## INTRODUCTION

Biological oscillations are essential for organisms to sense changing environments (e.g., nutrient-rich or -limiting growth conditions) and regulate their physiology according to new conditions (1). Saccharomyces cerevisiae, which is an extensively used model organism in industrial and medical applications, can exhibit self-sustaining oscillatory patterns in essential cellular processes like the cell division cycle (CDC), glucose metabolism, and respiration (2). These processes are regulated via rhythmic alterations in gene expression pattern and cell metabolism (1–3). The yeast metabolic cycle (YMC) and CDC are well-known examples of cellular oscillations. Such cycles are mediators for the comprehensive coordination of interconnected cellular activities (4, 5).

The YMC (also known as yeast respiratory oscillations and energy metabolism oscillations) is commonly characterized by respiratory oscillations in nutrient-limited chemostat cultures at high cell density (5). Even though several studies have reported that the carbon source limitation is not required to induce the YMC (4, 6), nutrient-limited chemostat cultivation is widely used to trigger self-synchronization of the oscillations in a yeast population. In this process, signal transduction plays an important role in the metabolic synchronization via dispersal of the secreted metabolites (5). Thus, the coordinated and dynamic nature of metabolic yeast behaviors can be retained through the generation of robust oscillations with a period of 4 to 5 h (2, 3, 5). The oscillation periods are dramatically affected by a variety of factors, such as strain type, culture condition, dilution rate, and oxygen level (2, 7). Based on the level of dissolved oxygen (dO_2_) in a yeast culture, two distinct YMC phases (growth-related high-oxygen-consumption [HOC] and stress/quiescence-related low-oxygen-consumption [LOC] phases) were described (3). In the LOC phase, yeast cells were reported to accumulate storage carbohydrates such as glycogen and trehalose to provide energy supplies for the HOC phase (1, 5). Differential transcript, chromatin accessibility, and metabolite profiles were reported over the course of these phases (6, 8–12). Robust periodic oscillations in gene expression allow subdivision of the HOC and LOC phases into three major stages, including the oxidative (OX), reductive building (RB), and reductive charging (RC) phases (2). In the quiescence-related RC phase, expression of the genes primarily involved in glycolysis and stress- and survival-related processes (e.g., stress resistance, heat shock, ubiquitination, and proteasomal degradation) are activated (2, 10, 13). NADPH and acetyl coenzyme A (acetyl-CoA) produced via the oxidation of fatty acids in this phase are transferred to the OX phase (9). NADPH acts as a buffering agent against oxidative stress due to the increasing oxygen consumption in the OX phase, while acetyl-CoA enables the acetylation of histones to induce growth-related genes (9, 13). Accordingly, expression of the genes associated with the synthesis of ribosomal proteins, amino acid metabolism, RNA processing, translation initiation factors, and sulfur metabolism are induced in the OX phase to prepare the cell for division in the next phase (2, 9, 13). While the intracellular oxygen levels decrease in the RB phase, the cell division-related genes encoding histone and spindle pole components as well as mitochondrial biogenesis-related genes are activated. The reduced oxygen consumption prevents potential DNA damage in the replication process (5, 9, 13–15). Overall, the YMC reflects the life cycle of yeast regulated by the interplay between growth, proliferation, and quiescent phases under nutrient-limited growth conditions, and timing of the DNA replication demonstrates the relationship between the CDC and YMC (1, 5, 14, 16). Thus, elucidation of the metabolic oscillations can provide great insight into the regulatory mechanisms acting on the growth programs of yeast cells (14).

Genome-scale metabolic network (GMN) models have been used as standard platforms to analyze yeast metabolism since 2003 (17). GMNs are mathematical representations of the cellular metabolism based on all known stoichiometry-based chemical reactions, metabolites, and their associated genes. Omics data-integrated yeast models have been reported to be beneficial in the characterization of yeast metabolism, phenotype predictions, and metabolic engineering studies under diverse growth conditions (18–22). Incorporation of different omics layers dramatically contributes to the understanding of the condition-specific cellular processes, and hence, this approach is used to develop next-generation genome-scale models of S. cerevisiae (23). The transcriptome is currently the most commonly used omics data to contextualize models due to its high accuracy and availability, compared to those of the proteome and metabolome (24, 25). At present, many useful approaches, such as GIMME (26), iMAT (27), INIT (28), E-Flux (29), PROM (30), and mCADRE (31), are available for the integration of the transcriptomic data in GMN models. In addition, several methods relying on combinatory use of constraint-based models and differential expression profiles (e.g., ΔFBA [32], MOOMIN [33], and REMI [34]) have been developed for the inference of metabolic changes from transcriptomic data. These methods are promising for condition-specific predictions on metabolic shifts without the need for specifying a biological objective function. Thus, existing alternative approaches facilitate the combinatory use of transcriptomics and metabolic modeling to represent cellular alterations under certain conditions. In addition to the transcriptomics, epigenetics also contributes to the understanding of cellular metabolism. Epigenetics is particularly promising due to its central role in shaping the gene expression profile without any changes in genome sequence. It is based on a set of combined modifications of DNA molecule and histone proteins (e.g., acetylation, methylation, and phosphorylation) that determine metabolic states under changing conditions (35, 36). Histone levels are strictly regulated in S. cerevisiae due to their widespread effect on transcriptional profile, and alterations in chromatin structure were reported to be crucial to determine differential expression levels. Nucleosome occupancy can be altered by transcriptional perturbations or histone-based epigenetic inheritance in the S phase (the phase of DNA replication) (37, 38). Various metabolic cofactors play a key role in shaping chromatin states via chromatin-modifying enzymes. Thus, fluctuations in metabolite levels are among the prominent factors regulating epigenome. Almost all chromatin-modifying enzymes need for metabolic cofactors. This relationship facilitates that metabolism-epigenome communication is an area of active research (39, 40). In this regard, the term metaboloepigenetics emerged to describe this reciprocal relationship (35, 41), and the potential link between diverse metabolic states and epigenetics in different biological systems has been reported (35, 42–44). For instance, the dependence of acetylation and methylation on acetyl-CoA and *S*-adenosylmethionine concentrations provides clear evidence for the relationship between epigenetic regulations and cell metabolism. The levels of chromatin-modifying metabolites are regulated via multiple mechanisms (e.g., nutrient availability), and metabolic regulation of the epigenome is essential to determine eukaryotic transcription levels (40). The cross talk between cellular metabolism and epigenetic modifications is essential to shape chromatin-mediated transcription profiles (35).

A positive correlation between transcriptome sequencing (RNA-seq) and transposase-accessible chromatin using sequencing (ATAC-seq) results was reported for a certain part of the genome (45). ATAC-seq is an efficient method developed in 2013 (46) to identify genome-wide chromatin accessibility. It is based on the use of hyperactive Tn*5* transposase enzymes to insert adapters into accessible (nucleosome-free) regions of the genome prior to high-throughput sequencing (47). There is a growing interest in ATAC-seq due to its superiorities over its counterparts (e.g., DNase I hypersensitive sites sequencing [DNase-seq] and formaldehyde-assisted isolation of regulatory elements sequencing [FAIRE-seq]), such as a requirement for a smaller sample size (500 to 50,000 cells), simpler/faster protocol, and a sensitivity and specificity higher than (or comparable to) those of its counterparts (47–49). Such epigenetic information can improve our insight into the cell metabolism by highlighting the effect of epigenetic modifications on the expression patterns of metabolic genes (35, 41), and combinatory approaches can expand the utilization of RNA-seq data in metabolic analyses. Despite the considerable effort to understand the relationship between epigenetics and metabolism, to our knowledge, the analysis of metabolic behaviors using ATAC-seq data in genome-scale metabolic modeling has not been reported yet. Importantly, genome-wide chromatin accessibility levels may provide additional information to represent diverse cellular states in an accurate manner and influence the predictive performances of GMN models. This prompted us to assess the capability of chromatin accessibility measurements for the prediction of varying cellular profile during the YMC in a comparative manner with RNA-seq data. We first characterized YMC phases using RNA-seq and ATAC-seq data sets. This enabled the evaluation of the data sets in terms of their ability to represent cellular phenotypes. We only considered metabolic genes in the current study and predicted YMC phenotypes in agreement with the previously reported studies. This indicates that variations between the phases could be successfully represented by both data sets. The phenotypic alterations during the YMC were also confirmed using two different model-based methods. In the first approach, we determined differential pathways consistent with data analysis results and literature. In the second approach, we simulated the YMC process through the contextualization of a generic GMN model using RNA-seq and ATAC-seq data sets. Performances of the context-specific models were evaluated using measured flux data sets across YMC phases, and more accurate results were obtained for the models integrated with chromatin accessibility data. Thus, we suggest that epigenetic information may be promising to shed light on dynamic metabolic cell behaviors.

## RESULTS AND DISCUSSION

In this study, we evaluated the contribution of epigenetic information to the prediction of metabolic profiles of S. cerevisiae across YMC phases. Chromatin accessibility and transcriptome levels derived from ATAC-seq and RNA-seq methods were used to achieve this goal. Integration of RNA-seq and microarray data sets into GMN models is an extensively used approach in condition-specific simulations. On the other hand, ATAC-seq data also provide a genome-wide information about dynamic cellular alterations. Therefore, we focused on the incorporation of this information in a generic yeast model for phenotype predictions across YMC phases.

**FIG 1:**
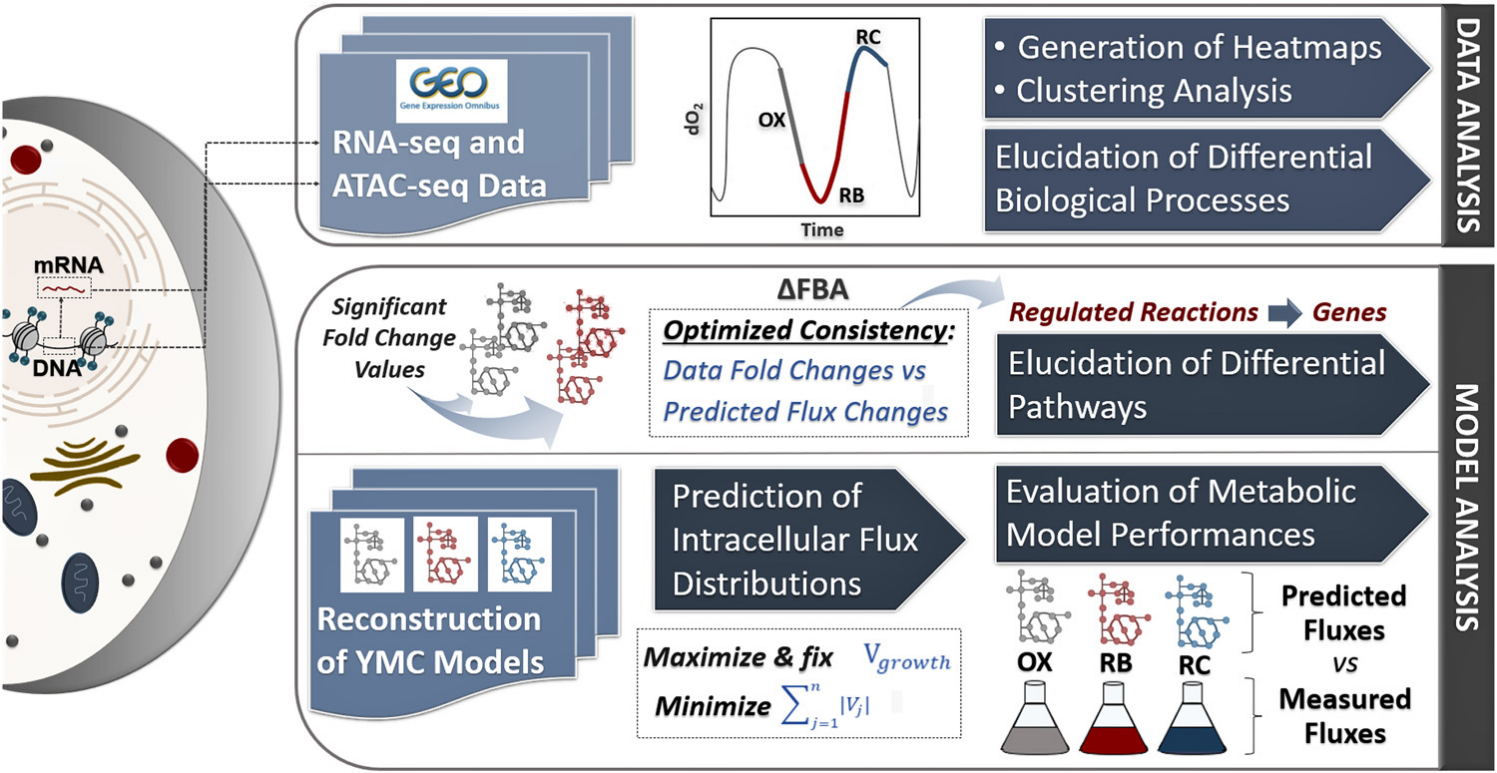
General flowchart for the analysis of ATAC-seq and RNA-seq data sets in addition to model-based analyses of the yeast metabolic cycle.

As summarized in the flowchart in Fig. 1, we first analyzed the chromatin accessibility and transcriptomic data sets consisting of six samples associated with YMC phases: RC (early and mid), OX (mid and late), and RB (early and late). For both data sets, we used hierarchical clustering on Yeast8 genes and selected three clusters according to the differential gene expression or chromatin accessibility levels. The biological processes in which clustered genes are involved were subsequently uncovered. In addition to data analysis, we performed model-based analyses to assess the potential use of ATAC-seq data in metabolic modeling. In this regard, two different approaches were used. First, we identified the reactions with differential fluxes based on chromatin accessibility changes. Pathways of the corresponding genes were uncovered during the transition from early RC phase to OX (mid and late) and RB (early and late) phases. Second, we reconstructed YMC models for three phases (early RC, mid OX, and late RB phases) through the GIMME algorithm considering gene expression and chromatin accessibility levels. The performances of these contextualized models were determined using experimentally measured fluxes. This also allowed us to evaluate the capability of ATAC-seq data in improving the model predictions (Fig. 1).

### Analysis of the RNA-seq and ATAC-seq data sets.

**FIG 2:**
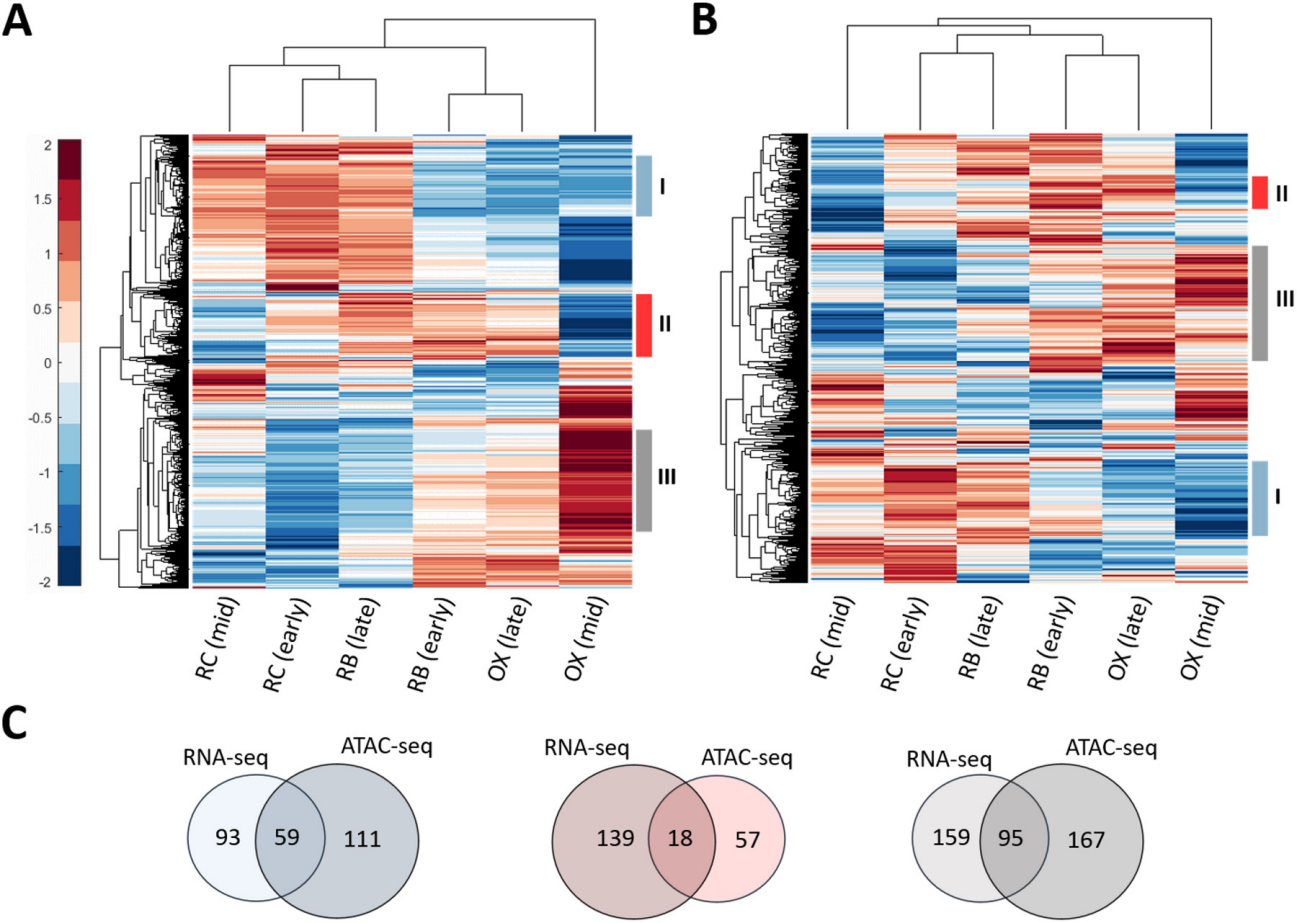
(A and B) Hierarchical clustering analysis of RNA-seq (A) and ATAC-seq (B) samples. Three gene clusters (designated I, II, and III) are selected based on the differential gene expression and chromatin accessibility levels. They are highlighted with bars in different colors. Clustered samples are also represented by the dendrograms. (C) Venn diagrams showing the numbers of common and distinct genes in each differentially expressed/accessible gene cluster.

Robust oscillations in the oxygen consumption trigger periodic changes in gene expression patterns, leading to oscillatory metabolic behaviors during the YMC (9, 10, 50). Therefore, characterization of the transcriptional and epigenetic regulations in the YMC process can provide important insight into the metabolic changes over time. We analyzed the RNA-seq and ATAC-seq data sets of wild-type S. cerevisiae strain CEN.PK in a comparative manner to examine the alterations in yeast metabolism across diverse YMC phases. We first mapped transcriptomic (see Table S1A in the supplemental material) and chromatin accessibility data sets (Table S1B) to the genes in the generic Yeast8 model. Using heat map graphs, we characterized the YMC-dependent cellular variations considering differential transcriptome and chromatin accessibility profiles across the YMC phases. We also performed hierarchical clustering to group the genes and samples based on their profiles. Thus, genes were grouped according to the phases in which they were predominantly upregulated. Three clusters, including phase-dependent upregulated genes (cluster I, RC [early and mid] and late RB; cluster II, RB [early and late] and late OX; and cluster III, early RB and OX [mid and late]) were selected for both RNA-seq (Fig. 2A) and ATAC-seq (Fig. 2B) data sets. In both data sets, the samples associated with early RC and late RB phases were found to be clustered together. Similarly, early RB and late OX phases share similar gene expression and chromatin accessibility patterns (Fig. 2A and B).

10.1128/msystems.01347-21.4 TABLE S1 (A) RNA-seq data sets matched with Yeast8 genes; (B) ATAC-seq data sets matched with Yeast8 genes. Download Table S1, XLSX file, 0.2 MB. Copyright © 2022 Cesur et al. 2022 Cesur et al. https://creativecommons.org/licenses/by/4.0/ This content is distributed under the terms of the Creative Commons Attribution 4.0 International license.

In RNA-seq data, cluster I includes 152 genes (Fig. 2C), which are predominantly upregulated in early RC phase. Gene Ontology (GO) enrichment analysis of this cluster indicated that these genes are mainly involved in lipid and carbohydrate metabolic processes (Table S2A). Tu and colleagues reported that fatty acid oxidation and breakdown of various storage carbohydrates are upregulated in the RC phase in order to contribute to available acetyl-CoA pool (8, 9). Accordingly, coenzyme metabolism was revealed to be enriched, as well. In ATAC-seq data, 170 genes in cluster I were found to be upregulated in particularly early RC phase. A total of 59 out of 170 genes were commonly identified through the analysis of RNA-seq data (Fig. 2C). They are mainly involved in oxidative stress response as well as lipid and cofactor metabolism. In fact, NADPH has a protective role against oxidative stress and its enhanced level was shown in the HOC phase (1). Previously, NADPH production was also reported in RC phase, and this metabolite is transferred into OX phase (9). Thus, oxidative stress response may be initiated during LOC-to-HOC transition. In a similar vein, we demonstrated that the NADP metabolic process was enriched for the 170 genes in cluster I. Furthermore, these genes were found to be associated with a set of biological processes such as carbohydrate (e.g., trehalose), alcohol, and coenzyme metabolism. As previously discussed, the accumulation of trehalose in the LOC phase is significant to fuel the energy production in the HOC phase. Trehalose has also protective roles during stress and starvation (1, 5, 51). Hence, an increase in the abundance of such carbohydrates was previously reported for the stress-related RC phase (8). In addition, it is not surprising to uncover alcohol metabolic process among overrepresented GO terms considering the reduced oxygen consumption in RC and late RB phases (Table S2B). Alcohol dehydrogenase and acetyl-CoA synthetase enzymes are induced in RC phase, and these mediate the production of acetyl-CoA, which is required for the activation of growth-related genes via acetylation in OX phase (9).

10.1128/msystems.01347-21.5 TABLE S2 Biological process enrichment analysis of the genes in cluster I using Gene Ontology Term Finder for RNA-seq data (A) and ATAC-seq data (B) (FDR at 0.01 level). Download Table S2, DOCX file, 0.03 MB. Copyright © 2022 Cesur et al. 2022 Cesur et al. https://creativecommons.org/licenses/by/4.0/ This content is distributed under the terms of the Creative Commons Attribution 4.0 International license.

In contrast to the quiescence-related RC phase, OX phase is characterized by a growth-related phenotype. Energy required to drive these processes is supplied by oxidative phosphorylation. In this regard, the acetyl-CoA produced in RC phase is transferred into OX phase and used in oxidative processes, including the tricarboxylic acid (TCA) cycle and electron transport chain for ATP production (9). The enhanced levels of acetyl-CoA and oxygen consumption lead to an elevated respiration rate in this phase (2, 7, 9). Indeed, we identified the overrepresentation of aerobic respiration for cluster II with 157 genes from RNA-seq data (Fig. 2C; Table S3A) and 75 genes from ATAC-seq data (Fig. 2C; Table S3B). In addition, acetyl-CoA plays a remarkable role in entry into the CDC by dynamically inducing histone acetylation for the expression of G_1_ cyclin CLN3. In the OX phase, the level of CLN3 peaks in response to fresh medium (52). Thus, acetyl-CoA-mediated histone acetylation can serve as a perfect bridge for the coordinated timing of growth (OX phase) and proliferation (RB phase) (13). In agreement with the growth-related profile of S. cerevisiae in OX phase, we demonstrated the overrepresentation of several fundamental biosynthetic processes, such as lipid, nucleotide, carbohydrate, and amino acid metabolism. These preparations are crucial to promote proliferation in the next phase. Similarly, the biosynthesis of amino acids (e.g., aspartate, lysine, and serine) and nucleotides were determined as enriched among the 95 common genes in cluster III, whose upregulations were shown in the differential analyses of both chromatin accessibility and gene expression profiles. This gene cluster represents upregulated genes in particularly mid OX phase: 254 genes for the RNA-seq data and 262 genes for the ATAC-seq data (Fig. 2C). In agreement with the overrepresented amino acid metabolism of cluster III genes (Table S4A and B), the elevated levels of lysine and serine in OX phase were previously reported by Tu and colleagues (8). In the translation process, such individual amino acids are delivered to the ribosome via aminoacyl-tRNAs (53). Accordingly, we demonstrated an enrichment for the metabolism and aminoacylation of tRNAs for protein synthesis. Another enriched process is *de novo* nucleotide synthesis. In OX phase, the overrepresentation of nucleotide metabolism under transcriptional control by Bas1 was reported. Bas1 is among the prominent regulators of the cell cycle in S. cerevisiae. This transcription factor regulates the synthesis of histidine, purines, and pyrimidines (2). Even if the relationship between Bas1 and the cell cycle is not clear (54), it may be significant to prepare the cells for division via the regulation of nucleotide and histidine pathways. Sulfur metabolism is another relevant process associated with the cell cycle. It is critical for the initiation of cell division in yeast (55). We identified this metabolic process to be enriched in OX phase, consistent with previous reports (7, 9). Thus, the analyses of RNA-seq and ATAC-seq data sets supported the literature-based evidence suggesting the activation of growth-related processes in OX phase to prepare yeast cells for cell division in RB phase (Table S4A and B).

10.1128/msystems.01347-21.6 TABLE S3 Biological process enrichment analysis of the genes in cluster II using Gene Ontology Term Finder for RNA-seq data (A) and ATAC-seq data (B) (FDR at 0.01 level). Download Table S3, DOCX file, 0.03 MB. Copyright © 2022 Cesur et al. 2022 Cesur et al. https://creativecommons.org/licenses/by/4.0/ This content is distributed under the terms of the Creative Commons Attribution 4.0 International license.

10.1128/msystems.01347-21.7 TABLE S4 Biological process enrichment analysis of the genes in cluster III using Gene Ontology Term Finder for RNA-seq data (A) and ATAC-seq data (B) (FDR at 0.01 level). Download Table S4, DOCX file, 0.04 MB. Copyright © 2022 Cesur et al. 2022 Cesur et al. https://creativecommons.org/licenses/by/4.0/ This content is distributed under the terms of the Creative Commons Attribution 4.0 International license.

### Model-based investigation of differential activity in yeast pathways.

Characterization of the differential metabolic genes through the analysis of both RNA-seq and ATAC-seq data sets was shown to be useful in order to gain a biological understanding of altered processes during progressive YMC phases. This encouraged us to further assess the power of ATAC-seq data. Therefore, we used this epigenomic information in metabolic modeling to examine phase-dependent metabolic changes. Using the ΔFBA approach, the Yeast8 reactions regulated by phase transitions were identified. This facilitated the elucidation of significantly altered KEGG pathways during the metabolic shift from early RC phase to OX and RB phases via corresponding genes.

**FIG 3:**
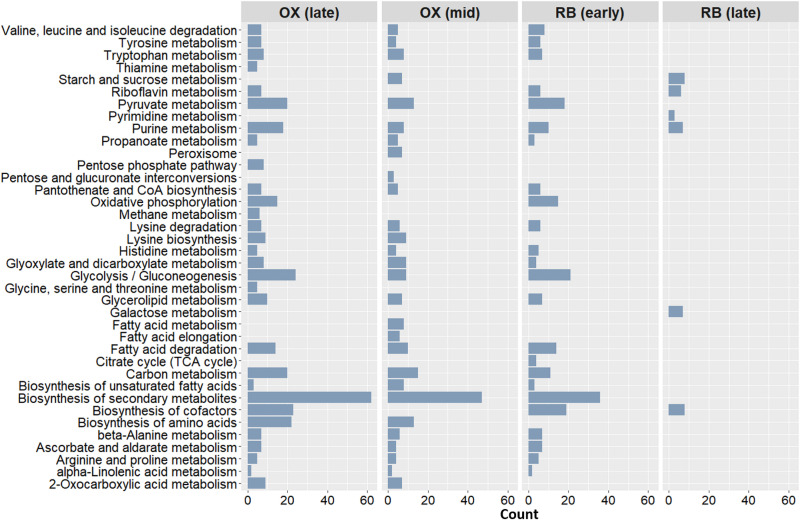
Bar plot of significantly enriched KEGG pathways that are regulated during the metabolic shift from early RC phase to OX (mid and late) and RB (early and late) phases. For each pathway, the horizontal axis (count) shows the number of the genes involved in that pathway.

The metabolic alterations in mid OX phase relative to early RC phase were first investigated. A total of 83 genes were identified in the regulated reactions. Similarly, 125 genes were uncovered to be associated with differential metabolism between early RC and late OX phases. As illustrated in Fig. 3, these OX-specific genes are predominantly involved in fundamental pathways (e.g., amino acid, fatty acid, carbohydrate, and purine metabolic pathways), in agreement with the growth-related phenotype of yeast. In addition, the pentose phosphate pathway (PPP) was found to be overrepresented in both mid (Table S5A) and late (Table S5B) OX phases. As explained in the data analysis part, amino acid synthesis is required to prepare yeast cells for cell division (2, 55). Aerobic respiration is another activated process in response to increasing oxygen consumption levels. Consistently, we determined the enrichment of oxidative phosphorylation in the late OX phase (Table S5B) similarly to the cluster II defined in the previous section (Fig. 2A and B). The changing oxygen intake also leads to regulation of the oxidative stress response. A variety of metabolites (e.g., homocysteine, serine, glutathione, and *S*-adenosylhomocysteine) participating in the regulatory pathways of sulfur metabolism were reported to display robust oscillations during the YMC (8). The sulfur-containing amino acids (methionine and cysteine) are synthesized from homocysteine in yeast (2, 56). Of these amino acids, cysteine can be subsequently used in the production of glutathione to avoid oxidative stress, which emerges due to varying oxygen consumption levels (8). Another key factor in glutathione production is NADPH. It is crucial for the conversion of sulfate to sulfide, which is important for the synthesis of homocysteine and hence glutathione (8, 56). NADPH can be produced by the oxidative branch of the PPP. Thus, this branch provides protection against oxidative stress, while the nonoxidative branch supports the biosynthesis of vital cellular components, including nucleic acids, fatty acids, and amino acids (57). The PPP was found to be overrepresented in mid (Table S5A) and (Table S5B) late OX phases. Importantly, we also determined enriched riboflavin metabolism in late OX phase (Table S5B) and RB phases (Table S5C and D). Its deficiency was reported to reduce glutathione reductase activity in liver cells. This leads to oxidative stress-induced DNA and protein damages as well as cell cycle arrest in G_1_ phase (58). Collectively, yeast cells appear to primarily concentrate on metabolic adaptation to the current nutritional status and preparation for the following phase. Consistently, the overrepresentation of carbon flow through oxidative phosphorylation and TCA cycle continues in the early RB phase for the adaptation into periodic oscillations in the oxygen consumption (Table S5C). Thus, this metabolic shift toward higher oxidative phosphorylation activity can also allow a decline in dissolved oxygen level for both establishment of characteristic features in the early RB phase and the initiation of cell division in the late next phase (Fig. 3; Table S5C and D) (2, 10).

10.1128/msystems.01347-21.8 TABLE S5 KEGG pathway enrichment analysis of the genes in the altered reactions during transition from early RC phase to mid OX phase (A), from early RC phase to late OX phase (B), from early RC phase to early RB phase (C), and from early RC phase to late RB phase (D) using the g:Profiler web server for ATAC-seq data (FDR at 0.05 level). Download Table S5, DOCX file, 0.03 MB. Copyright © 2022 Cesur et al. 2022 Cesur et al. https://creativecommons.org/licenses/by/4.0/ This content is distributed under the terms of the Creative Commons Attribution 4.0 International license.

In early and late RB phases, we revealed coordinated regulation of nucleotide metabolism, carbon metabolism, riboflavin metabolism, and cofactor synthesis (Fig. 3; Table S5C and D). As highlighted above, the regulation of amino acid biosynthesis and oxidative phosphorylation are maintained in early RB phase, indicating the importance of metabolic alterations for the adaptation between consecutive phases (Table S5C). The metabolic relationship between early RB phase and late OX phase is consistent with the observed clustering profiles of these phases. At early stages of the proliferation-related RB phase, a similar expression profile with late OX phase is exhibited (Fig. 2A and B). We identified fewer genes (*n* = 46) associated with the regulated pathways in the late RB phase than in early RB phase (*n* = 89) and OX phases (*n* = 83 [mid] and *n* = 125 [late]). Differential chromatin accessibility and gene expression patterns also confirmed this result. As described in the previous section, early RC and late RB phases were clustered together (Fig. 2A and B). Thus, we did not expect extensive differences between their metabolic profiles. Unsurprisingly, carbohydrate metabolism and nucleotide metabolism were shown to be enriched in the late RB phase (Fig. 3; Table S5D). Carbon metabolism is especially prominent for the glycosylation process and cell wall integrity, which are required for viability and appropriate cell division. For instance, defective O-mannosylation was reported to cause a reduction in yeast growth (59). We could successfully predict the dynamic metabolic status of the YMC phases via the differential ATAC-seq data-based analysis of the Yeast8 model. The metabolic predictions were found to be consistent with data analysis results (see previous section) and literature. Therefore, these findings indicated that genome-wide epigenetic information holds promise for metabolic modeling.

### Model-based analysis of differential flux profiles.

**FIG 4:**
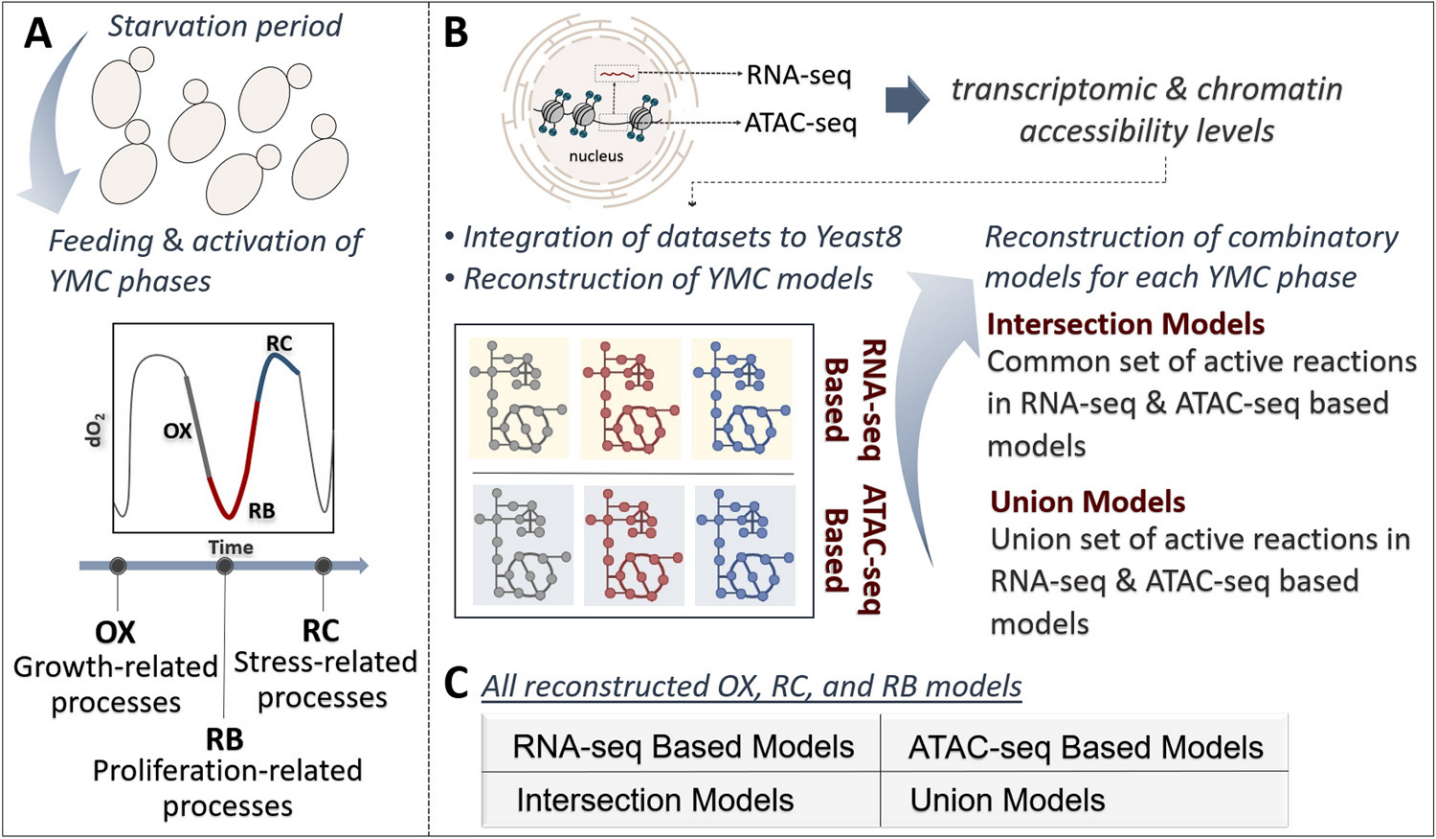
Reconstruction process of the contextualized models. (A) Feeding-aided induced YMC phases (growth-related OX phase, proliferation-related RB phase, and stress/quiescence-related RC phase). (B) Incorporation of RNA-seq or ATAC-seq data sets dedicated to each YMC phase into the Yeast8 model allowed the reconstruction of RNA-seq- and ATAC-seq-based yeast models. Using the reaction activity information in these models, intersection and union models were also reconstructed for each YMC phase. (C) Thus, each YMC phase is represented by four diverse models.

To further confirm the impact of chromatin accessibility data on metabolic predictions, we also used this epigenetic knowledge in the contextualization of the generic yeast model. In this regard, we investigated the efficiency of these data for the prediction of differential flux profiles and assessed their predictive capacity in a comparative manner with RNA-seq data. Context-specific GMN models have been extensively used to characterize yeast metabolism (18–22). Therefore, we surveyed altered yeast phenotype through the reconstruction of YMC models. Using the GIMME algorithm (threshold of 25th percentile), we generated four different GMN models (RNA-seq-based, ATAC-seq-based, intersection, and union models) for each selected phase (early RC, mid OX, and late RB) (Fig. 4). Of these models, intersection models were created according to the reactions that are active in both RNA-seq-based and ATAC-seq-based models, while union models were created considering the reactions active in either model. Maximum growth rates were predicted to be between ~0.10 h^−1^ and ~0.12 h^−1^ for all models simulated in minimal medium, and this range of values is consistent with the measured growth rate of the aerobic chemostat S. cerevisiae culture (60). The contents of the YMC models are presented in Table 1.

**TABLE 1:** Four different models developed for each YMC phase using transcriptomic and chromatin accessibility data sets^*a*^

Model	No. of:			YMC phase
	Reactions	Metabolites	Genes	
RNA-seq-based model	3,161	2,431	931	Early RC
	3,010	2,360	903	Mid OX
	3,215	2,446	960	Late RB
ATAC-seq-based model	3,338	2,526	968	Early RC
	3,295	2,515	951	Mid OX
	3,354	2,532	971	Late RB
Intersection model	2,763	2,323	835	Early RC
	2,543	2,214	803	Mid OX
	2,796	2,314	848	Late RB
Union model	3,737	2,619	1,065	Early RC
	3,765	2,608	1,057	Mid OX
	3,774	2,648	1,084	Late RB

a The numbers of reactions, metabolites, and genes involved in YMC models are listed.

We evaluated the performances of the reconstructed YMC models based on the metabolic fluxes measured by Zhang and colleagues (60). They investigated dynamic metabolic flux distributions associated with major carbon utilization pathways across diverse CDC stages (G_0_/G_1_ entry, G_0_/G_1_ phase, and late G_1_/S phase transition). A clear relationship between metabolic cycling and G_0_/G_1_ phase was reported for slowly growing budding yeasts, while transcriptional cycling was also identified in G_2_ phase for fission yeasts (50). To explain the relationship between the CDC and YMC, a simple YMC-CDC coupling model has been proposed. It is based on gating of the CDC by metabolic cycles in different yeast strains and under distinct nutrient conditions in order to coordinate the timing of cellular growth and division (4, 61, 62). The CDC requires the accumulation of sufficient energy sources to reach a critical cell size, and OX phase enables achievement of this commitment threshold known as “Start.” Thus, yeast cells irreversibly commit to the CDC through the expression of hundreds of G_1_/S genes (5, 10). As explained before, CLN3 is among the activated growth-associated genes in OX phase. It regulates the length of G_1_ phase and cell size for transition into S phase (14, 52, 63). DNA replication and cell division were proposed to start at a very late stage of the OX phase and continue in RB phase under a relatively small amount of the intracellular oxygen (9, 10, 13). These phenomena occur once per YMC (5). The relationship between the YMC and CDC oscillators allowed us to use the measured flux data set obtained by Zhang and colleagues (60) for evaluation of the YMC models.

**FIG 5:**
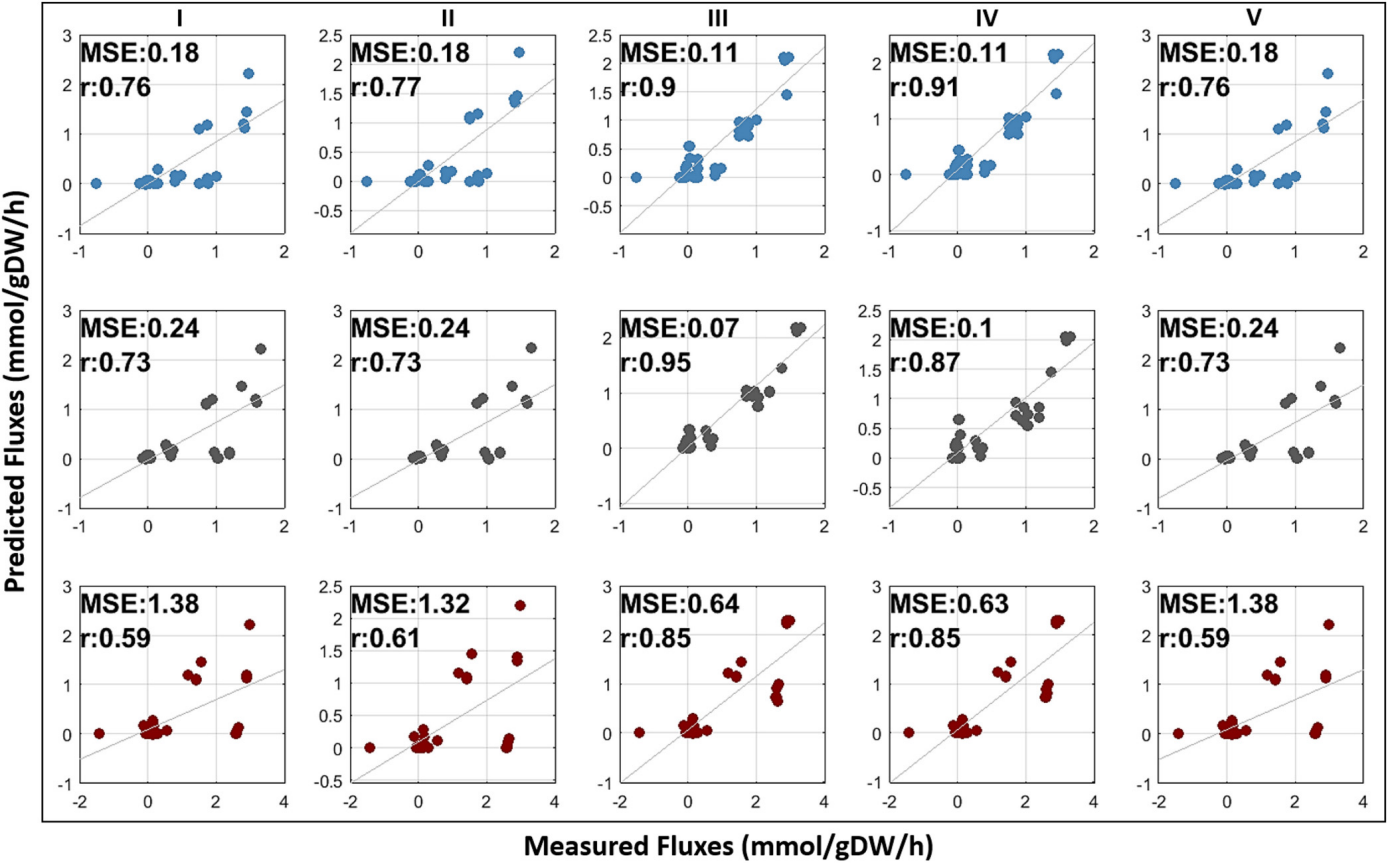
Comparison of the predicted and measured flux distributions for different GMN models (I, generic Yeast8 model; II, RNA-seq-based model; III, ATAC-seq-based model; IV, intersection model; V, union model) across early RC, mid OX, and late RB phases (colored blue, gray, and red). Mean squared error (MSE) and Pearson’s correlation coefficient (*r*) are shown for each model.

Prior to the use of measured fluxes, we matched the YMC phases and sampling time points of flux measurements according to dO_2_ levels. We identified three time points overlapped with early RC, mid OX, and late RB phases. At least 37 reactions with measured fluxes were used to assess the flux prediction performances of YMC models (Table S6B). Phase-specific flux maps were first created for RNA-seq- and ATAC-seq-based models. Increased glycolytic flux into TCA cycle in OX phase was shown through the ATAC-seq-based model in Fig. S1 to S3, which is conceivable considering the elevated oxygen consumption levels during OX phase, as previously highlighted. The experimental and predicted fluxes of the yeast reactions were subsequently compared for all models (Table 1) through Pearson’s correlation coefficients and mean squared errors (MSE) to measure the capability of the models in the prediction of differential flux profiles in a comparative manner (Fig. 5). According to both metrics, integration of the chromatin accessibility data sets dramatically improved the model predictions, with higher correlation (*r* = 0.85 to 0.95) and predominantly lower error values (MSE = 0.07 to 0.64), compared to the generic Yeast8 model (*r* = 0.59 to 0.76 and MSE = 0.18 to 1.38) and RNA-seq-based YMC models (*r* = 0.61 to 0.77 and MSE = 0.18 to 1.32). The significance of the differences between the correlation coefficients was evaluated using two-sample z test at a *P* value cutoff of 0.05. Apart from the RC phase (*P* value = 0.065), significant correlation differences were found between ATAC-seq- and RNA-seq-based models (OX phase *P* value = 0.0005 and RB phase *P* value = 0.023). Furthermore, integration of ATAC-seq data resulted in significant correlation differences in comparison with the generic Yeast8 model for all YMC phases. Flux prediction capacity was also evaluated for the combinatory YMC models, which were contextualized by both transcriptomic and chromatin accessibility data. Fluxes from intersection models also exhibited higher correlation to experimental values (*r* = 0.85 to 0.91) than those from RNA-seq-based models. These correlation values were shown to be significantly different at RC phase (*P* value = 0.031) and RB phase (*P* value = 0.019) from those of the RNA-seq-based models. Similarly, the correlation differences were found to be significant (RC phase *P* value = 0.022 and RB phase *P* value = 0.013) in comparison with Yeast8 models, although we did not find a significant difference between the performances of OX models (*P* value = 0.088) at the significance level of 0.05. On the other hand, we did not observe considerable significant differences between the correlation values calculated for the union models and Yeast8 model. This indicates that the union models are less powerful to represent the condition-specific behavior of yeast cells.

10.1128/msystems.01347-21.1 FIG S1 Estimated flux values of central carbon metabolism through RNA-seq-based (A) and ATAC-seq-based (B) metabolic network models. Both flux maps correspond to early reductive charging (RC) phase, and flux values are given in millimoles per gDW per hour. The dashed arrows show blocked (inactive) reactions due to the preference of alternative routes. Abbreviations for the Embden-Meyerhof pathway: Glc, glucose; G6P, glucose-6-phosphate; F6P, fructose-6-phosphate; FBP, fructose-1,6-bisphosphate; DHAP, dihydroxyacetone phosphate; GAP, glyceraldehyde-3-phosphate; G3P, glycerol 3-phosphate; PG3, 3-phosphoglycerate; PG2, 2-phosphoglycerate; PEP, phosphoenolpyruvate; PYR, pyruvate. Abbreviations for the trehalose biosynthesis pathway: T6P, trehalose 6-phosphate; Treh, trehalose. Abbreviations for the glycogen biosynthesis pathway: G1P, glucose 1-phosphate; UDPG, uridine diphosphate glucose; Glyco, glycogen. Abbreviations for the pentose phosphate pathway: PG6, 6-phosphogluconate; Ribu5P, ribulose-5-phosphate; X5P, xylulose 5-phosphate; Rib5P, ribose-5-phosphate; S7P, sedoheptulose-7-phosphate; E4P, erythrose-4-phosphate. Abbreviations for the tricarboxylic acid cycle: AcCoA, acetyl coenzyme A; Cit, citrate; iCit, isocitrate; AKG, α-ketoglutarate; Succ, succinate; Fum, fumarate; Mal, malate; OAA, oxaloacetate. Abbreviation for the glyoxylate bypass: gliox, glyoxylate. Abbreviations for the amino acid synthesis pathway: Ala, alanine; Asp, aspartate; Glu, glutamate. Download FIG S1, TIF file, 0.5 MB. Copyright © 2022 Cesur et al. 2022 Cesur et al. https://creativecommons.org/licenses/by/4.0/ This content is distributed under the terms of the Creative Commons Attribution 4.0 International license.

10.1128/msystems.01347-21.9 TABLE S6 (A) Intracellular fluxes measured by ^13^C-MFA are mapped to the reactions in the Yeast8 model. (B) Intracellular fluxes measured by ^13^C-MFA are mapped to the active reactions in all YMC models (RNA-seq-based, ATAC-seq-based, intersection, and union models) for each phase. Flux values are given in millimoles per gDW per hour. Download Table S6, DOCX file, 0.03 MB. Copyright © 2022 Cesur et al. 2022 Cesur et al. https://creativecommons.org/licenses/by/4.0/ This content is distributed under the terms of the Creative Commons Attribution 4.0 International license.

Compared to RNA-seq-based models, chromatin accessibility further supports to generate accurate YMC models for the inference of carbon metabolism (Fig. 5). To confirm the effect of these data sets on metabolic flux predictions, we also reconstructed YMC models using additional threshold values (35th, 45th, and 50th percentiles) in the GIMME algorithm (Table S7). ATAC-seq data improved the capability of models to predict intracellular distributions regardless of the selected thresholds, leading to higher correlation and lower MSE values for both ATAC-seq-based models and intersection models. Collectively, the contextualization of Yeast8 models via chromatin accessibility data sets extensively increased the power of yeast models for the system-wide quantification of intracellular fluxes across diverse YMC phases.

10.1128/msystems.01347-21.10 TABLE S7 Comparison of the predicted and measured fluxes for different thresholds used in the GIMME algorithm (objective fraction: 0.5). Each column is colored by comparing the corresponding metrics within itself. A color scale is used from blue (lower values) to white (higher values) to represent the magnitude of mean squared errors, while white (lower values) to red (higher values) scale is used for Pearson’s correlation coefficients. Download Table S7, DOCX file, 0.04 MB. Copyright © 2022 Cesur et al. 2022 Cesur et al. https://creativecommons.org/licenses/by/4.0/ This content is distributed under the terms of the Creative Commons Attribution 4.0 International license.

**FIG 6:**
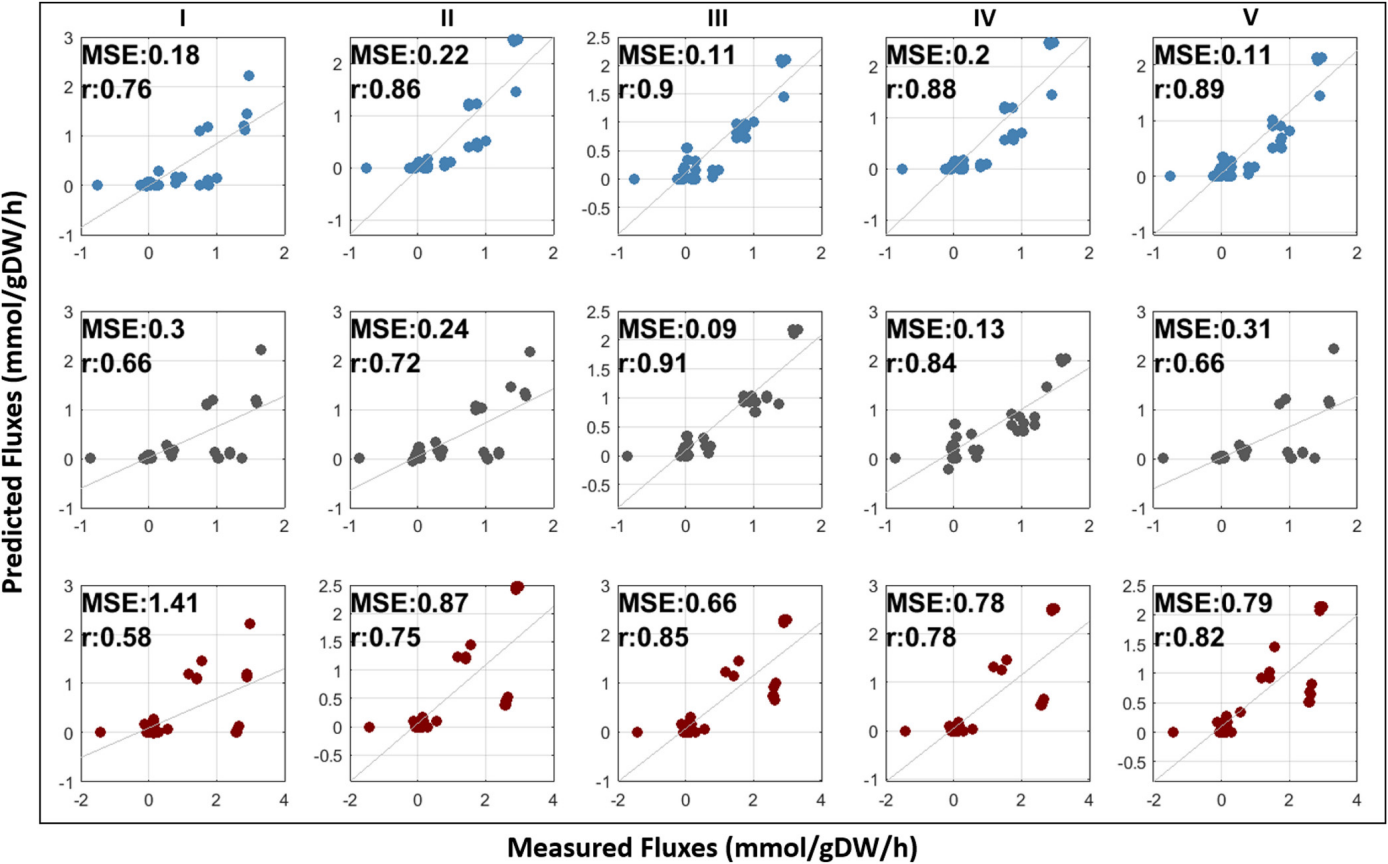
Comparison of the predicted and measured flux distributions for different GMN models based on filtered data (I, generic Yeast8 model; II, RNA-seq-based model; III, ATAC-seq-based model; IV, intersection model; V, union model) across early RC, mid OX, and late RB phases (colored blue, gray, and red). Mean squared error (MSE) and Pearson’s correlation coefficient (*r*) are shown for each model.

A subset of the genes in RNA-seq data set that were not measured in ATAC-seq data set. To test if the difference between the measured gene compositions in RNA-seq and ATAC-seq data sets plays a role in the differences observed in the metabolic predictions, we removed the genes that were measured only in RNA-seq data set, and we repeated the analyses with this dataset. This allowed us to consider only commonly measured genes of both data sets in the model reconstruction process. Using the filtered data sets, four distinct YMC models were developed across each YMC phase (early RC, mid OX, and late RB phases) (Table 2). We subsequently examined the model performances to detect whether the superiority of chromatin accessibility data in terms of flux correlations is due to the variations in the measured gene sets. Despite an increase in the flux prediction capability of RNA-seq-based models (*r* = 0.72 to 0.86 and MSE = 0.22 to 0.87), incorporation of the chromatin accessibility levels was shown to result in higher correlation to the experimental measurements once more in both ATAC-seq-based models (*r* = 0.85 to 0.91) and intersection models (*r* = 0.78 to 0.88). For the filtered data sets, union models also exhibited considerably better correlation values than did RNA-seq-based models for RC and RB phases as a result of more stringent constraints imposed by the filtered data. Furthermore, the integration of chromatin accessibility data sets led to a reduction in MSE values in comparison with gene expression data sets (Fig. 6). Overall, chromatin accessibility information allowed more accurate estimation of diverse metabolic states for the same measured gene sets, as well.

**TABLE 2:** Four different models developed for each YMC phase using filtered transcriptomic and chromatin accessibility data sets^*a*^

Model	No. of:			YMC phase
	Reactions	Metabolites	Genes	
RNA-seq-based model	3,209	2,440	936	Early RC
	3,074	2,362	923	Mid OX
	3,218	2,449	953	Late RB
ATAC-seq-based model	3,338	2,526	968	Early RC
	3,295	2,515	951	Mid OX
	3,354	2,532	971	Late RB
Intersection model	2,833	2,337	853	Early RC
	2,619	2,218	830	Mid OX
	2,836	2,330	859	Late RB
Union model	3,716	2,612	1,053	Early RC
	3,753	2,605	1,050	Mid OX
	3,738	2,631	1,067	Late RB

a The numbers of reactions, metabolites, and genes involved in YMC models are listed.

Although it is not intuitive to find a higher-level cellular regulation layer (chromosome accessibility) to better represent a lower layer (metabolism), rather than an intermediate layer (RNA-seq), there may be several reasons for this outcome. One reason may be the lower signal/noise ratio in RNA-seq data than in ATAC-seq data, leading to less accurate metabolic models. Or, the success of ATAC-seq data in the network contextualization for dynamic events may be explained by the fact that mRNA levels reflect a pool of molecules produced in a certain time period, whereas the ATAC-seq profile reflects the momentary regulation state of the gene at a specific time point. Hence, the ATAC-seq profile may be more accurate reflection of the cell state at a given time point. For instance, ATAC-seq and RNA-seq patterns were reported to differ in the S/G_2_ phase of the CDC for embryonic stem cells (64) even if a strong correlation between transcriptome and chromatin accessibility data sets was observed for the housekeeping genes in eukaryotic cells (45). This phenomenon was validated through the analysis of protein-coding genes by showing the weak coupling between the dynamics of chromatin architecture and transcriptional induction during early differentiation. Alterations in chromatin organization were suggested to occur rapidly during differentiation to prevent alternative cell fate decisions in the CDC (64). Similarly, Liu and colleagues demonstrated that the combinatory use of steady-state expression level and chromatin status is beneficial to understand the profiles of periodically regulated genes and regulatory mechanisms in breast cancer cells during the CDC (65).

Nevertheless, this preliminary work requires further validation for especially higher eukaryotic organisms. Even though S. cerevisiae provides remarkable information on the epigenetic processes underlying the establishment and maintenance of histone modification-dependent chromatin states in higher eukaryotes, this organism has a relatively simple epigenetic system without many mammalian histone variants, repressive histone mark (H3K9 methylation), DNA methylation, and RNA interference machinery (37, 66, 67). Yeast cells also lack the complicated cross talk between the DNA methylation and histone modification pathways, unlike animal cells (66, 67). Therefore, ATAC-seq data-guided model analyses should be applied for higher eukaryotes to assess the comprehensive utilization of such data sets. In this regard, available methods for the simultaneous measurement of DNA accessibility and gene expression dynamics (68–70) and the increasing popularity of integrative analysis of ATAC-seq and RNA-seq data sets (71–73) may facilitate further evaluation of the efficacy of ATAC-seq data in future metabolic modeling studies.

### Conclusion.

ATAC-seq is a recent technology to understand epigenetic changes based on genome-wide chromatin accessibility (46, 74). It can be easily applied for diverse cell types and tissues with a lower cost and sample volume (74). These advantages support the higher popularity of ATAC-seq data sets in large genomics consortiums (e.g., ENCODE [75], TCGA [76], and TaRGET [77]) than other technologies associated with genome-wide chromatin accessibility profiling (e.g., DNase-seq, micrococcal nuclease sequencing [MNase-seq], and FAIRE-seq) (74).

Incorporation of the gene expression profiles to GMN models is a powerful approach to characterize condition-specific metabolic behaviors of organisms. On the other hand, there is still a gap in metabolic modeling to use the dynamic epigenetic information on gene expression regulation, which is provided by ATAC-seq technique (78). The relationship between epigenetics and cell metabolism encouraged us to investigate the effect of ATAC-seq data in GMN-based prediction of cell metabolism. Therefore, we first confirmed the successful application of these epigenetic data in metabolic modeling through the prediction of differential yeast pathways. In addition, we evaluated the contribution of chromatin accessibility information to the characterization of the YMC metabolism in terms of differential flux profiles. Contextualization of the generic yeast model by ATAC-seq data sets was shown to enhance the predictive performance of the models in comparison with RNA-seq data-integrated models. In other words, ATAC-seq-based models exhibited performances superior to those of the other context-specific models (i.e., RNA-seq-based models and combinatory models) reconstructed in the scope of this study.

Taken together, the results lead us to propose that accessible chromatin states may provide promising insight for the discovery of altered dynamic metabolic processes during metabolic cycles. In other words, the elucidation of dynamic epigenetic landscapes may be remarkable for a deeper understanding of variations in cell metabolism.

## MATERIALS AND METHODS

### Metabolic network model.

We used the recent consensus genome-scale metabolic network model of S. cerevisiae, Yeast8, consisting of 1,147 genes and 2,691 metabolites which take part in a total of 3,991 reactions (79). This compartmentalized model is publicly available via GitHub (https://github.com/SysBioChalmers/yeast-GEM/releases/tag/v8.3.5). Growth-associated maintenance, referring to the energy required for biomass formation, was set to 55.3 mmol ATP/g dry weight (gDW). Non-growth-associated maintenance responsible for the energy dedicated to cellular functions apart from growth was used as 0.7 mmol ATP/gDW/h (79). This model allowed identification of the metabolic genes in RNA-seq and ATAC-seq data sets in addition to the simulation of YMC phases as explained in the following sections.

### Analysis of the RNA-seq and ATAC-seq data sets.

The RNA-seq and ATAC-seq data sets (GEO accession number GSE101290) that were generated and processed by Gowans and colleagues for wild-type S. cerevisiae strain CEN.PK (10) were used in the current study. The researchers generated the robust recurring oscillations of the YMC by following the protocol in the study of Tu et al. (9). They measured gene expression and chromatin accessibility levels across three YMC phases. Samples from two time points were collected for each phase: (i) early and mid RC, (ii) mid and late OX, and (iii) early and late RB (10). Each time point has two biological replicates, and we used the averages of replicates per time point. We subsequently mapped the measured gene expression and chromatin accessibility data sets to Yeast8 genes (Table S1). To investigate the relationship among YMC phases, we applied hierarchical clustering to the log-transformed data based on Euclidean distance between the samples via the *clustergram* function in MATLAB R2017b and also generated heat maps. Considering the differential expression/chromatin accessibility profiles, we selected three subclusters including upregulated genes in diverse YMC phases: (i) RC (early and mid) and late RB, (ii) RB (early and late) and late OX, and (iii) early RB and OX (mid and late). The clustered genes were characterized through the investigation of the related biological processes using GO Term Finder tool (version 0.86) (80) provided by *Saccharomyces* Genome Database (81) at a false-discovery rate (FDR) of 0.01 (Tables S2 to S4).

### Model-based investigation of differential activity in yeast pathways.

In the first part of the model analysis, we investigated differential activity in yeast pathways based on ATAC-seq data. To do so, chromatin accessibility fold changes of gene promoter regions were calculated relative to the control (early RC phase) for OX (mid and late) and RB (early and late) phases. For each phase, we discarded the genes with no significant alterations in their accessibility levels (FDR [also known as *q* value] ≥ 0.01), which were identified through R (version 4.1.0) limma-trend (default settings) (82). Using the *mapExpressionToReactions* function of COBRA Toolbox (83) in MATLAB 2017b, these filtered fold change values were mapped to the reactions in the Yeast8 model via gene-protein-reaction (GPR) associations. This function assigned minimum gene accessibility fold change to a reaction if the corresponding genes were linked with the “AND” operator, whereas maximum gene accessibility fold change was assigned for the genes linked with the “OR” operator. We used a recent approach, ΔFBA, in order to identify flux changes during metabolic shifts. This approach is based on a two-step optimization procedure that maximizes the consistency and minimizes inconsistency (L2-norm minimization) between flux changes (Δv) and the mapped differential accessibility levels (32). Thus, this method does not need *a priori* knowledge of a cellular objective such as biomass formation or ATP production (32). We predicted Δv distributions for both OX and RB phases in comparison with early RC phase. The altered reaction sets showing flux changes above a relaxed threshold value (ε = 0.1) were determined. The corresponding genes in these reactions were uncovered via GPR rules. Significantly enriched KEGG pathways associated with these genes were identified using g:Profiler web server (84) with FDR at a 0.05 level.

### Reconstruction of the YMC models.

In the second part of model analysis, we evaluated the contribution of RNA-seq and ATAC-seq data sets to the predictive power of the Yeast8 in terms of differential flux patterns. The GIMME algorithm was used to generate the context-specific GMN models representing YMC phases (here referred to as YMC models) through the integration of transcriptome and chromatin accessibility data sets to the generic Yeast8 model. Distinct models were built for three time points (early RC, mid OX, and late RB phases) using RNA-seq and ATAC-seq data sets (Fig. 4A and B; Table 1). To do so, the data sets were first mapped to the reactions in Yeast8 based on GPR rules via the COBRA *mapExpressionToReactions* function. In the GIMME algorithm, the gene levels mapped to the reactions are used as an input. Other inputs are threshold value, GMN model, and objective fraction (26). We applied the threshold of the 25th percentile for the identification of active/inactive genes in the Yeast8 model for each data set. GIMME solves an optimization problem relying on mass balance constraints. In this regard, it minimizes the number of reactions corresponding to lowly expressed genes below the threshold while maximizing the number of the reactions in the model associated with active genes (26). We set the objective fraction value to 0.5, which allows growth rate to decrease to 50% of its model-based maximum value.

In addition to the RNA-seq-based and ATAC-seq-based models (early RC, mid OX, and late RB models) reconstructed as described above, we developed mass-balanced combinatory models (intersection and union models) using multi-omics information. To this end, we generated a binary vector to represent the active/inactive state of reactions. Reactions that were active in either of the ATAC-seq and RNA-seq models were represented as 1 in this binary vector for the union model, while the rest of reactions were set to zero. Similarly, another binary vector was created to generate intersection models such that only reactions active in both ATAC-seq and RNA-seq models were represented as 1 (Fig. 4B; Table 1). These binary vectors were used as input to the GIMME algorithm with a threshold value of 0.5, which ensured that the algorithm tried to keep reactions represented with 1 in the model while trying to remove the reactions represented with 0 from the model by satisfying mass balance constraints. Overall, a total of four different YMC models (i.e., RNA-seq-based, ATAC-seq-based, intersection, and union models) were developed for each phase (Fig. 4C).

We repeated the model reconstruction steps for filtered data sets to test the impact of measured data compositions on the performances of the YMC models. In the RNA-seq data set, there are genes whose levels were not detected in the ATAC-seq data sets. We removed these genes from the data sets. Then, the filtered data sets were integrated to the generic Yeast8 model using the GIMME algorithm as explained above. In other words, only commonly measured genes were considered in the model reconstruction process. Thus, an additional model was developed for each YMC phase. These models are listed in Table 2. As highlighted, this step is significant to investigate whether the difference between model performances is due to the variations in sizes of the measured gene sets (see the following section).

### Model-based analysis of differential flux profiles.

YMC models were simulated under minimal medium (9, 85) for the glucose uptake rate of ~1.45 mmol/gDW//h (60). Flux balance analysis (FBA) is the most common constraint-based modeling approach to simulate particularly microbial metabolism at steady state (86, 87). Using the FBA approach, we simulated growth profiles to identify intracellular flux distributions. In the optimization step, two different objective functions were used consecutively to represent yeast metabolism in an accurate manner. In the first optimization problem, we aimed the maximization of cellular growth because this biological objective was reported to represent the best fit with experimental data (88) and it is widely used to simulate microbial metabolism in FBA modeling (89, 90). The maximum growth rate predicted in the optimization step was subsequently constrained by allowing its 10% reduction, and minimization of the sum of absolute intracellular fluxes was introduced as the secondary objective to narrow down solution space (91). The FBA approach is formulated as follows:

(1) $$\overset{n}{\underset{j=1}{\sum }}\left(S_{\mathrm{ij}}V_{j}\right)=0\;$$

(2) $${\text{lb}}_{j}\;\leq \;V_{j}\;\leq \;{\text{ub}}_{j}$$

(3) $$\text{maximize }V_{\text{growth}}\left(\text{1st objective function}\right)$$

(4) $$\;0.9\times V_{\text{opt}}\;\leq \;V_{\text{growth}}\;\leq \;V_{\text{opt}}$$

(5) $$\text{minimize}\sum_{j=1}^{n}\left|V_{j}\right|\left(\text{2nd objective function}\right)$$

Equation 1 indicates the mass balance around each metabolite at steady state, where the stoichiometric matrix represented as *S_ij_* with the coefficient of metabolite *i* within a total of *m* metabolites, and *V_j_* represents the flux of the *j*th reaction within a total of *n* reactions. In addition to the assumption on mass conservation, an assumption on the reversibility of biochemical reactions is used in the FBA approach. Accordingly, lb*_j_* and ub*_j_* are the flux boundaries (lower and upper bounds) for the *j*th reaction as shown in equation 2. Equation 3 demonstrates the optimization step used for the prediction of optimal growth rate (*V*_opt_), which satisfies the given primary objective function. Predicted growth rate was used as a constraint in the next step. In equation 4, we allowed the growth rate to be flexible by 10% of *V*_opt_. A secondary optimization (minimization of the enzyme production) was subsequently applied based on the minimization of sum of absolute intracellular fluxes as formulated in equation 5 (91). Here, we solved the optimization problems using Gurobi solver (version 8.0.1). All simulations were performed for both the generic Yeast8 model and YMC models using MATLAB R2017b.

### Validation of differential flux profiles.

To evaluate the capacity of YMC models in the prediction of flux distributions, we compared predicted fluxes with the measured counterparts derived from ^13^C metabolic flux analysis (MFA) for S. cerevisiae strain CEN.PK117-5D (60). Similar to the study of Gowans and colleagues (10), the minimal cultivation condition described by Tu et al. (9) was used to obtain the measured fluxes. Hence, cultivation conditions of the ATAC-seq/RNA-seq data (10) and flux data (60) were the same. Using the ^13^C-MFA method, Zhang and colleagues examined the metabolic transition between CDC phases via additional glucose intake by the synchronized yeast cells, and reaction fluxes were determined for selected sampling points (60). Here, we first matched the flux sampling points and YMC phases. Cycling oxygen level is a prominent factor to coordinate the timing of cellular growth and division (4, 61, 62). Therefore, we used oxygen oscillation as a gold standard to ensure a reliable matching between samples. Accordingly, we considered the changing dO_2_ levels reported in the ^13^C-MFA study (60) and the RNA-seq/ATAC-seq study (10). Thus, overlapped time points between both studies were detected in terms of dO_2_ profiles. The matched time points correspond to early RC, mid OX, and late RB phases. Hence, flux comparisons were merely applied for these three phases. To do so, we mapped 43 experimentally detected fluxes (60) to the reactions in the generic Yeast8 model for each YMC phase (Table S6A). At least 37 out of 43 matched reactions were found to be shared across yeast models (RNA-seq-based, ATAC-seq-based, intersection, and union models) for each YMC phase (Table S6B). For these reactions, we compared the experimental and predicted fluxes via two metrics: Pearson’s correlation coefficient (*r*) and mean squared error (MSE). We also compared the significance of the differences between correlation coefficients. Using Fisher’s z transformation, each correlation coefficient was first converted to z-score, which is approximately normally distributed (92, 93). Then, test statistic was calculated to compare the correlation coefficients (z-scores) of samples based on two-sample z test (93). We identified significantly different correlation coefficient values (*P* value cutoff, 0.05).

10.1128/msystems.01347-21.2 FIG S2 Estimated flux values of the central carbon metabolism through RNA-seq-based (A) and ATAC-seq-based (B) metabolic network models. Both flux maps correspond to mid oxidative (OX) phase, and flux values are given in millimoles per gDW per hour. The dashed arrows show blocked (inactive) reactions due to the preference of alternative routes. Abbreviations are as for Fig. S1. Download FIG S2, TIF file, 0.5 MB. Copyright © 2022 Cesur et al. 2022 Cesur et al. https://creativecommons.org/licenses/by/4.0/ This content is distributed under the terms of the Creative Commons Attribution 4.0 International license.

10.1128/msystems.01347-21.3 FIG S3 Estimated flux values of the central carbon metabolism through RNA-seq-based (A) and ATAC-seq-based (B) metabolic network models. Both flux maps correspond to late reductive building (RB) phase, and flux values are given in millimoles per gDW per hour. The dashed arrows show blocked (inactive) reactions due to the preference of alternative routes. Abbreviations are as for Fig. S1. Download FIG S3, TIF file, 0.6 MB. Copyright © 2022 Cesur et al. 2022 Cesur et al. https://creativecommons.org/licenses/by/4.0/ This content is distributed under the terms of the Creative Commons Attribution 4.0 International license.
