# Supplementary material for: Genome-Wide Analysis of Yeast Metabolic Cycle through Metabolic Network Models Reveals Superiority of Integrated ATAC-seq Data over RNA-seq Data
Source: mSystems. 2022 Jun 13;7(3):e01347-21. doi: 10.1128/msystems.01347-21 (PMC9239220; doi:10.1128/msystems.01347-21)
Supplement: TABLE S2 [file msystems.01347-21-st002.docx]

**Table S2A**

| **GO ID** | **Term name** | **Adjusted p-value** | **Number of genes** |
| --- | --- | --- | --- |
| GO:0044281 | small molecule metabolic process | 1.30E-30 | 70 |
| GO:0043436 | oxoacid metabolic process | 1.26E-22 | 46 |
| GO:0006082 | organic acid metabolic process | 1.43E-22 | 46 |
| GO:0019752 | carboxylic acid metabolic process | 1.57E-22 | 45 |
| GO:0032787 | monocarboxylic acid metabolic process | 1.45E-21 | 31 |
| GO:0044282 | small molecule catabolic process | 7.44E-20 | 26 |
| GO:0044242 | cellular lipid catabolic process | 1.43E-14 | 15 |
| GO:0016042 | lipid catabolic process | 2.51E-14 | 15 |
| GO:0051186 | cofactor metabolic process | 3.45E-14 | 29 |
| GO:0072329 | monocarboxylic acid catabolic process | 4.32E-14 | 15 |
| GO:0044255 | cellular lipid metabolic process | 5.04E-13 | 32 |
| GO:0016054 | organic acid catabolic process | 6.83E-13 | 18 |
| GO:0046395 | carboxylic acid catabolic process | 6.83E-13 | 18 |
| GO:0009062 | fatty acid catabolic process | 1.47E-12 | 11 |
| GO:0006629 | lipid metabolic process | 1.94E-12 | 32 |
| GO:0006631 | fatty acid metabolic process | 1.86E-10 | 14 |
| GO:0055114 | oxidation-reduction process | 4.24E-10 | 22 |
| GO:0006732 | coenzyme metabolic process | 4.84E-10 | 21 |
| GO:1901575 | organic substance catabolic process | 4.99E-10 | 41 |
| GO:0006635 | fatty acid beta-oxidation | 2.59E-09 | 8 |
| GO:0046496 | nicotinamide nucleotide metabolic process | 1.52E-08 | 14 |
| GO:0019395 | fatty acid oxidation | 1.95E-08 | 8 |
| GO:0034440 | lipid oxidation | 1.95E-08 | 8 |
| GO:0019362 | pyridine nucleotide metabolic process | 2.39E-08 | 14 |
| GO:0009117 | nucleotide metabolic process | 3.40E-08 | 20 |
| GO:0055086 | nucleobase-containing small molecule metabolic process | 4.10E-08 | 22 |
| GO:0006733 | oxidoreduction coenzyme metabolic process | 4.70E-08 | 15 |
| GO:0019637 | organophosphate metabolic process | 6.02E-08 | 28 |
| GO:0006753 | nucleoside phosphate metabolic process | 7.63E-08 | 20 |
| GO:0072524 | pyridine-containing compound metabolic process | 1.25E-07 | 14 |
| GO:0044283 | small molecule biosynthetic process | 4.29E-07 | 24 |
| GO:0009056 | catabolic process | 6.60E-07 | 43 |
| GO:0006979 | response to oxidative stress | 1.58E-06 | 15 |
| GO:0071704 | organic substance metabolic process | 2.18E-06 | 104 |
| GO:0008152 | metabolic process | 2.21E-06 | 107 |
| GO:0017144 | drug metabolic process | 2.47E-06 | 19 |
| GO:0006091 | generation of precursor metabolites and energy | 4.99E-06 | 17 |
| GO:1901135 | carbohydrate derivative metabolic process | 6.73E-06 | 23 |
| GO:0034599 | cellular response to oxidative stress | 3.08E-05 | 13 |
| GO:0030258 | lipid modification | 3.21E-05 | 9 |
| GO:0005975 | carbohydrate metabolic process | 5.33E-05 | 18 |
| GO:0016052 | carbohydrate catabolic process | 8.38E-05 | 11 |
| GO:0044237 | cellular metabolic process | 1.23E-04 | 101 |
| GO:0006793 | phosphorus metabolic process | 1.80E-04 | 33 |
| GO:0006090 | pyruvate metabolic process | 1.88E-04 | 8 |
| GO:0019693 | ribose phosphate metabolic process | 2.31E-04 | 13 |
| GO:1901615 | organic hydroxy compound metabolic process | 2.85E-04 | 13 |
| GO:0005996 | monosaccharide metabolic process | 3.46E-04 | 11 |
| GO:0009987 | cellular process | 1.01E-03 | 119 |
| GO:0019318 | hexose metabolic process | 1.16E-03 | 10 |
| GO:0006081 | cellular aldehyde metabolic process | 3.00E-03 | 6 |
| GO:0042737 | drug catabolic process | 3.94E-03 | 6 |
| GO:0051188 | cofactor biosynthetic process | 4.18E-03 | 12 |
| GO:0006688 | glycosphingolipid biosynthetic process | 4.55E-03 | 3 |
| GO:0006796 | phosphate-containing compound metabolic process | 5.61E-03 | 29 |
| GO:0046031 | ADP metabolic process | 6.50E-03 | 6 |
| GO:0015980 | energy derivation by oxidation of organic compounds | 8.07E-03 | 12 |
| GO:0009199 | ribonucleoside triphosphate metabolic process | 8.08E-03 | 9 |
| GO:0009135 | purine nucleoside diphosphate metabolic process | 8.21E-03 | 6 |
| GO:0009179 | purine ribonucleoside diphosphate metabolic process | 8.21E-03 | 6 |
| GO:0009185 | ribonucleoside diphosphate metabolic process | 8.21E-03 | 6 |

**Table S2B**

| **GO ID** | **Term name** | **Adjusted p-value** | **Number of genes** |
| --- | --- | --- | --- |
| GO:0044281 | small molecule metabolic process | 2.40E-23 | 66 |
| GO:0051186 | cofactor metabolic process | 1.08E-13 | 30 |
| GO:0043436 | oxoacid metabolic process | 1.53E-12 | 37 |
| GO:0006082 | organic acid metabolic process | 1.68E-12 | 37 |
| GO:0019752 | carboxylic acid metabolic process | 2.05E-12 | 36 |
| GO:0044282 | small molecule catabolic process | 2.95E-09 | 18 |
| GO:0055114 | oxidation-reduction process | 4.95E-09 | 22 |
| GO:0032787 | monocarboxylic acid metabolic process | 2.11E-08 | 20 |
| GO:0044255 | cellular lipid metabolic process | 1.19E-07 | 27 |
| GO:0008152 | metabolic process | 2.20E-07 | 120 |
| GO:0006732 | coenzyme metabolic process | 3.16E-07 | 19 |
| GO:0006629 | lipid metabolic process | 3.40E-07 | 27 |
| GO:0071704 | organic substance metabolic process | 1.10E-06 | 115 |
| GO:0051188 | cofactor biosynthetic process | 1.57E-06 | 17 |
| GO:0044237 | cellular metabolic process | 1.71E-06 | 116 |
| GO:0005975 | carbohydrate metabolic process | 2.33E-06 | 21 |
| GO:0055086 | nucleobase-containing small molecule metabolic process | 2.56E-06 | 21 |
| GO:0019637 | organophosphate metabolic process | 4.58E-06 | 27 |
| GO:0044242 | cellular lipid catabolic process | 2.56E-05 | 9 |
| GO:0016054 | organic acid catabolic process | 2.78E-05 | 12 |
| GO:0046395 | carboxylic acid catabolic process | 2.78E-05 | 12 |
| GO:0016042 | lipid catabolic process | 3.41E-05 | 9 |
| GO:0006979 | response to oxidative stress | 6.46E-05 | 14 |
| GO:1901135 | carbohydrate derivative metabolic process | 6.63E-05 | 23 |
| GO:1901615 | organic hydroxy compound metabolic process | 1.87E-04 | 14 |
| GO:0006575 | cellular modified amino acid metabolic process | 1.98E-04 | 9 |
| GO:0006790 | sulfur compound metabolic process | 2.91E-04 | 14 |
| GO:0016052 | carbohydrate catabolic process | 3.02E-04 | 11 |
| GO:0009117 | nucleotide metabolic process | 4.14E-04 | 16 |
| GO:0046942 | carboxylic acid transport | 4.70E-04 | 11 |
| GO:0015718 | monocarboxylic acid transport | 5.17E-04 | 7 |
| GO:0015849 | organic acid transport | 5.42E-04 | 11 |
| GO:0072329 | monocarboxylic acid catabolic process | 6.52E-04 | 8 |
| GO:0006631 | fatty acid metabolic process | 6.80E-04 | 9 |
| GO:0006753 | nucleoside phosphate metabolic process | 7.50E-04 | 16 |
| GO:0009062 | fatty acid catabolic process | 9.14E-04 | 6 |
| GO:0006091 | generation of precursor metabolites and energy | 9.99E-04 | 15 |
| GO:0015980 | energy derivation by oxidation of organic compounds | 1.07E-03 | 14 |
| GO:0006066 | alcohol metabolic process | 1.22E-03 | 11 |
| GO:0006733 | oxidoreduction coenzyme metabolic process | 1.57E-03 | 11 |
| GO:0009311 | oligosaccharide metabolic process | 1.57E-03 | 8 |
| GO:1901575 | organic substance catabolic process | 1.71E-03 | 32 |
| GO:0009108 | coenzyme biosynthetic process | 1.77E-03 | 11 |
| GO:0006811 | ion transport | 1.99E-03 | 20 |
| GO:0044262 | cellular carbohydrate metabolic process | 2.02E-03 | 14 |
| GO:0006793 | phosphorus metabolic process | 2.91E-03 | 33 |
| GO:0009987 | cellular process | 3.81E-03 | 130 |
| GO:0072524 | pyridine-containing compound metabolic process | 4.16E-03 | 10 |
| GO:0046365 | monosaccharide catabolic process | 4.39E-03 | 5 |
| GO:0005992 | trehalose biosynthetic process | 5.69E-03 | 4 |
| GO:0009312 | oligosaccharide biosynthetic process | 5.69E-03 | 4 |
| GO:0046351 | disaccharide biosynthetic process | 5.69E-03 | 4 |
| GO:0034599 | cellular response to oxidative stress | 6.16E-03 | 11 |
| GO:0006739 | NADP metabolic process | 6.70E-03 | 5 |
| GO:0046496 | nicotinamide nucleotide metabolic process | 8.52E-03 | 9 |
| GO:0042737 | drug catabolic process | 8.66E-03 | 6 |
