# Supplementary material for: Genome-Wide Analysis of Yeast Metabolic Cycle through Metabolic Network Models Reveals Superiority of Integrated ATAC-seq Data over RNA-seq Data
Source: mSystems. 2022 Jun 13;7(3):e01347-21. doi: 10.1128/msystems.01347-21 (PMC9239220; doi:10.1128/msystems.01347-21)
Supplement: TABLE S3 [file msystems.01347-21-st003.docx]

**Table S3A**

| **GO ID** | **Term name** | **Adjusted p-value** | **Number of genes** |
| --- | --- | --- | --- |
| GO:0044281 | small molecule metabolic process | 2.14E-48 | 88 |
| GO:0009205 | purine ribonucleoside triphosphate metabolic process | 6.49E-30 | 30 |
| GO:0046034 | ATP metabolic process | 6.49E-30 | 30 |
| GO:0009123 | nucleoside monophosphate metabolic process | 1.28E-29 | 32 |
| GO:0009144 | purine nucleoside triphosphate metabolic process | 1.90E-29 | 30 |
| GO:0009199 | ribonucleoside triphosphate metabolic process | 1.90E-29 | 30 |
| GO:0009126 | purine nucleoside monophosphate metabolic process | 2.75E-29 | 31 |
| GO:0009141 | nucleoside triphosphate metabolic process | 2.75E-29 | 31 |
| GO:0009167 | purine ribonucleoside monophosphate metabolic process | 2.75E-29 | 31 |
| GO:0009161 | ribonucleoside monophosphate metabolic process | 7.02E-29 | 31 |
| GO:0009150 | purine ribonucleotide metabolic process | 1.71E-28 | 32 |
| GO:0019637 | organophosphate metabolic process | 2.40E-28 | 51 |
| GO:0006163 | purine nucleotide metabolic process | 8.51E-28 | 32 |
| GO:0009259 | ribonucleotide metabolic process | 8.51E-28 | 32 |
| GO:0072521 | purine-containing compound metabolic process | 5.36E-27 | 33 |
| GO:0019693 | ribose phosphate metabolic process | 1.77E-25 | 32 |
| GO:0009117 | nucleotide metabolic process | 4.58E-25 | 36 |
| GO:0055086 | nucleobase-containing small molecule metabolic process | 5.80E-25 | 39 |
| GO:0006753 | nucleoside phosphate metabolic process | 2.42E-24 | 36 |
| GO:1901135 | carbohydrate derivative metabolic process | 4.45E-23 | 43 |
| GO:0017144 | drug metabolic process | 1.27E-20 | 34 |
| GO:0045333 | cellular respiration | 1.24E-18 | 24 |
| GO:0006793 | phosphorus metabolic process | 1.99E-18 | 55 |
| GO:0015980 | energy derivation by oxidation of organic compounds | 3.61E-18 | 28 |
| GO:0008152 | metabolic process | 5.57E-18 | 130 |
| GO:0019752 | carboxylic acid metabolic process | 3.50E-17 | 40 |
| GO:0006091 | generation of precursor metabolites and energy | 1.54E-16 | 28 |
| GO:0006119 | oxidative phosphorylation | 1.83E-16 | 15 |
| GO:0022900 | electron transport chain | 1.83E-16 | 15 |
| GO:0022904 | respiratory electron transport chain | 1.83E-16 | 15 |
| GO:0042773 | ATP synthesis coupled electron transport | 1.83E-16 | 15 |
| GO:0042775 | mitochondrial ATP synthesis coupled electron transport | 1.83E-16 | 15 |
| GO:0043436 | oxoacid metabolic process | 2.03E-16 | 40 |
| GO:0006082 | organic acid metabolic process | 2.26E-16 | 40 |
| GO:0006796 | phosphate-containing compound metabolic process | 3.44E-16 | 51 |
| GO:0055114 | oxidation-reduction process | 6.84E-16 | 28 |
| GO:0044237 | cellular metabolic process | 1.37E-15 | 125 |
| GO:0090407 | organophosphate biosynthetic process | 2.57E-15 | 29 |
| GO:0006754 | ATP biosynthetic process | 5.92E-14 | 12 |
| GO:0009145 | purine nucleoside triphosphate biosynthetic process | 5.92E-14 | 12 |
| GO:0009206 | purine ribonucleoside triphosphate biosynthetic process | 5.92E-14 | 12 |
| GO:0015985 | energy coupled proton transport, down electrochemical gradient | 5.92E-14 | 12 |
| GO:0015986 | ATP synthesis coupled proton transport | 5.92E-14 | 12 |
| GO:0009060 | aerobic respiration | 6.74E-14 | 18 |
| GO:0009142 | nucleoside triphosphate biosynthetic process | 6.85E-14 | 13 |
| GO:0071704 | organic substance metabolic process | 8.20E-14 | 121 |
| GO:0009201 | ribonucleoside triphosphate biosynthetic process | 3.87E-13 | 12 |
| GO:0009124 | nucleoside monophosphate biosynthetic process | 4.89E-13 | 14 |
| GO:1901566 | organonitrogen compound biosynthetic process | 8.84E-13 | 53 |
| GO:0009127 | purine nucleoside monophosphate biosynthetic process | 2.04E-12 | 13 |
| GO:0009168 | purine ribonucleoside monophosphate biosynthetic process | 2.04E-12 | 13 |
| GO:1901564 | organonitrogen compound metabolic process | 2.29E-12 | 81 |
| GO:0009156 | ribonucleoside monophosphate biosynthetic process | 6.29E-12 | 13 |
| GO:1902600 | hydrogen ion transmembrane transport | 1.45E-11 | 12 |
| GO:0009152 | purine ribonucleotide biosynthetic process | 2.07E-11 | 14 |
| GO:0006164 | purine nucleotide biosynthetic process | 4.54E-11 | 14 |
| GO:0015992 | proton transport | 4.67E-11 | 12 |
| GO:0015672 | monovalent inorganic cation transport | 7.05E-11 | 13 |
| GO:0009260 | ribonucleotide biosynthetic process | 9.51E-11 | 14 |
| GO:0070127 | tRNA aminoacylation for mitochondrial protein translation | 1.75E-10 | 8 |
| GO:1901293 | nucleoside phosphate biosynthetic process | 1.82E-10 | 17 |
| GO:0046390 | ribose phosphate biosynthetic process | 6.93E-10 | 14 |
| GO:0072522 | purine-containing compound biosynthetic process | 6.93E-10 | 14 |
| GO:0009987 | cellular process | 7.27E-10 | 135 |
| GO:0009058 | biosynthetic process | 1.23E-09 | 77 |
| GO:1901137 | carbohydrate derivative biosynthetic process | 1.35E-09 | 23 |
| GO:0009165 | nucleotide biosynthetic process | 1.63E-09 | 16 |
| GO:0043038 | amino acid activation | 1.93E-09 | 12 |
| GO:0043039 | tRNA aminoacylation | 1.93E-09 | 12 |
| GO:1901576 | organic substance biosynthetic process | 2.16E-09 | 76 |
| GO:0098660 | inorganic ion transmembrane transport | 2.22E-09 | 14 |
| GO:0098662 | inorganic cation transmembrane transport | 2.89E-09 | 13 |
| GO:0044249 | cellular biosynthetic process | 2.05E-07 | 71 |
| GO:0006418 | tRNA aminoacylation for protein translation | 3.63E-07 | 10 |
| GO:0044238 | primary metabolic process | 4.35E-07 | 104 |
| GO:0098655 | cation transmembrane transport | 4.45E-07 | 13 |
| GO:0006122 | mitochondrial electron transport, ubiquinol to cytochrome c | 6.82E-07 | 7 |
| GO:0034220 | ion transmembrane transport | 1.11E-06 | 15 |
| GO:0072350 | tricarboxylic acid metabolic process | 1.45E-06 | 7 |
| GO:0006520 | cellular amino acid metabolic process | 1.79E-06 | 20 |
| GO:0032543 | mitochondrial translation | 2.84E-06 | 13 |
| GO:0044255 | cellular lipid metabolic process | 1.08E-05 | 23 |
| GO:0032787 | monocarboxylic acid metabolic process | 1.42E-05 | 16 |
| GO:0006629 | lipid metabolic process | 2.56E-05 | 23 |
| GO:0006123 | mitochondrial electron transport, cytochrome c to oxygen | 3.71E-05 | 6 |
| GO:0019646 | aerobic electron transport chain | 3.71E-05 | 6 |
| GO:0006812 | cation transport | 6.92E-05 | 15 |
| GO:0008610 | lipid biosynthetic process | 8.48E-05 | 17 |
| GO:0044283 | small molecule biosynthetic process | 9.04E-05 | 21 |
| GO:0006644 | phospholipid metabolic process | 1.03E-04 | 15 |
| GO:0006811 | ion transport | 1.03E-04 | 21 |
| GO:0140053 | mitochondrial gene expression | 1.12E-04 | 13 |
| GO:0046486 | glycerolipid metabolic process | 1.32E-04 | 14 |
| GO:0006650 | glycerophospholipid metabolic process | 1.78E-04 | 13 |
| GO:0006101 | citrate metabolic process | 4.86E-04 | 5 |
| GO:0000959 | mitochondrial RNA metabolic process | 6.83E-04 | 8 |
| GO:0033615 | mitochondrial proton-transporting ATP synthase complex assembly | 8.76E-04 | 5 |
| GO:0043461 | proton-transporting ATP synthase complex assembly | 8.76E-04 | 5 |
| GO:0070272 | proton-transporting ATP synthase complex biogenesis | 8.76E-04 | 5 |
| GO:0008654 | phospholipid biosynthetic process | 1.17E-03 | 11 |
| GO:0043648 | dicarboxylic acid metabolic process | 1.34E-03 | 6 |
| GO:0055085 | transmembrane transport | 1.97E-03 | 18 |
| GO:0072330 | monocarboxylic acid biosynthetic process | 2.14E-03 | 8 |
| GO:0051186 | cofactor metabolic process | 3.06E-03 | 16 |
| GO:0046474 | glycerophospholipid biosynthetic process | 4.04E-03 | 9 |
| GO:0045017 | glycerolipid biosynthetic process | 7.58E-03 | 9 |

**Table S3B**

| **GO ID** | **Term name** | **Adjusted p-value** | **Number of genes** |
| --- | --- | --- | --- |
| GO:1901135 | carbohydrate derivative metabolic process | 9.30E-13 | 23 |
| GO:0019637 | organophosphate metabolic process | 2.89E-12 | 24 |
| GO:0072521 | purine-containing compound metabolic process | 8.57E-11 | 15 |
| GO:0044281 | small molecule metabolic process | 2.89E-10 | 30 |
| GO:0009123 | nucleoside monophosphate metabolic process | 9.92E-10 | 13 |
| GO:0009205 | purine ribonucleoside triphosphate metabolic process | 1.51E-09 | 12 |
| GO:0046034 | ATP metabolic process | 1.51E-09 | 12 |
| GO:0009144 | purine nucleoside triphosphate metabolic process | 2.16E-09 | 12 |
| GO:0009199 | ribonucleoside triphosphate metabolic process | 2.16E-09 | 12 |
| GO:0009126 | purine nucleoside monophosphate metabolic process | 8.09E-09 | 12 |
| GO:0009141 | nucleoside triphosphate metabolic process | 8.09E-09 | 12 |
| GO:0009167 | purine ribonucleoside monophosphate metabolic process | 8.09E-09 | 12 |
| GO:0017144 | drug metabolic process | 1.01E-08 | 16 |
| GO:0009161 | ribonucleoside monophosphate metabolic process | 1.10E-08 | 12 |
| GO:0055086 | nucleobase-containing small molecule metabolic process | 3.75E-08 | 16 |
| GO:0009150 | purine ribonucleotide metabolic process | 4.52E-08 | 12 |
| GO:0006163 | purine nucleotide metabolic process | 7.57E-08 | 12 |
| GO:0009259 | ribonucleotide metabolic process | 7.57E-08 | 12 |
| GO:0006796 | phosphate-containing compound metabolic process | 1.61E-07 | 25 |
| GO:0009117 | nucleotide metabolic process | 2.12E-07 | 14 |
| GO:0006753 | nucleoside phosphate metabolic process | 3.80E-07 | 14 |
| GO:0006793 | phosphorus metabolic process | 4.18E-07 | 25 |
| GO:0019693 | ribose phosphate metabolic process | 4.31E-07 | 12 |
| GO:1901137 | carbohydrate derivative biosynthetic process | 1.05E-06 | 14 |
| GO:0090407 | organophosphate biosynthetic process | 1.13E-06 | 14 |
| GO:1901564 | organonitrogen compound metabolic process | 1.28E-06 | 41 |
| GO:1901566 | organonitrogen compound biosynthetic process | 6.84E-06 | 26 |
| GO:0006629 | lipid metabolic process | 1.22E-05 | 16 |
| GO:0034220 | ion transmembrane transport | 2.27E-05 | 10 |
| GO:0006811 | ion transport | 2.49E-05 | 15 |
| GO:0006812 | cation transport | 3.89E-05 | 11 |
| GO:0044255 | cellular lipid metabolic process | 4.49E-05 | 15 |
| GO:0008152 | metabolic process | 1.26E-04 | 57 |
| GO:0044237 | cellular metabolic process | 1.59E-04 | 56 |
| GO:0071704 | organic substance metabolic process | 2.53E-04 | 55 |
| GO:0072522 | purine-containing compound biosynthetic process | 2.86E-04 | 7 |
| GO:0009124 | nucleoside monophosphate biosynthetic process | 3.11E-04 | 6 |
| GO:0006754 | ATP biosynthetic process | 3.36E-04 | 5 |
| GO:0009145 | purine nucleoside triphosphate biosynthetic process | 3.36E-04 | 5 |
| GO:0009206 | purine ribonucleoside triphosphate biosynthetic process | 3.36E-04 | 5 |
| GO:0015985 | energy coupled proton transport, down electrochemical gradient | 3.36E-04 | 5 |
| GO:0015986 | ATP synthesis coupled proton transport | 3.36E-04 | 5 |
| GO:0009201 | ribonucleoside triphosphate biosynthetic process | 5.98E-04 | 5 |
| GO:0044238 | primary metabolic process | 7.87E-04 | 52 |
| GO:0006091 | generation of precursor metabolites and energy | 1.02E-03 | 10 |
| GO:1901576 | organic substance biosynthetic process | 1.07E-03 | 36 |
| GO:0009142 | nucleoside triphosphate biosynthetic process | 1.27E-03 | 5 |
| GO:0009058 | biosynthetic process | 1.42E-03 | 36 |
| GO:0046486 | glycerolipid metabolic process | 1.51E-03 | 9 |
| GO:0005975 | carbohydrate metabolic process | 1.76E-03 | 11 |
| GO:1902600 | hydrogen ion transmembrane transport | 1.97E-03 | 5 |
| GO:0044249 | cellular biosynthetic process | 2.04E-03 | 35 |
| GO:0055085 | transmembrane transport | 2.41E-03 | 12 |
| GO:0045017 | glycerolipid biosynthetic process | 2.64E-03 | 7 |
| GO:0015992 | proton transport | 2.94E-03 | 5 |
| GO:0009127 | purine nucleoside monophosphate biosynthetic process | 3.55E-03 | 5 |
| GO:0009168 | purine ribonucleoside monophosphate biosynthetic process | 3.55E-03 | 5 |
| GO:0006644 | phospholipid metabolic process | 4.23E-03 | 9 |
| GO:0008610 | lipid biosynthetic process | 4.40E-03 | 10 |
| GO:0009165 | nucleotide biosynthetic process | 4.62E-03 | 7 |
| GO:0006650 | glycerophospholipid metabolic process | 4.83E-03 | 8 |
| GO:0009156 | ribonucleoside monophosphate biosynthetic process | 5.06E-03 | 5 |
| GO:1901293 | nucleoside phosphate biosynthetic process | 5.51E-03 | 7 |
| GO:0045333 | cellular respiration | 8.35E-03 | 7 |
