# Supplementary material for: Genome-Wide Analysis of Yeast Metabolic Cycle through Metabolic Network Models Reveals Superiority of Integrated ATAC-seq Data over RNA-seq Data
Source: mSystems. 2022 Jun 13;7(3):e01347-21. doi: 10.1128/msystems.01347-21 (PMC9239220; doi:10.1128/msystems.01347-21)
Supplement: TABLE S4 [file msystems.01347-21-st004.docx]

**Table S4A**

| **GO ID** | **Term name** | **Adjusted p-value** | **Number of genes** |
| --- | --- | --- | --- |
| GO:0044281 | small molecule metabolic process | 2.04E-62 | 126 |
| GO:0043436 | oxoacid metabolic process | 1.46E-57 | 93 |
| GO:0006082 | organic acid metabolic process | 1.95E-57 | 93 |
| GO:0019752 | carboxylic acid metabolic process | 7.38E-55 | 89 |
| GO:0006520 | cellular amino acid metabolic process | 1.25E-53 | 71 |
| GO:1901566 | organonitrogen compound biosynthetic process | 2.41E-53 | 124 |
| GO:0016053 | organic acid biosynthetic process | 7.58E-44 | 57 |
| GO:0046394 | carboxylic acid biosynthetic process | 7.58E-44 | 57 |
| GO:0008652 | cellular amino acid biosynthetic process | 2.20E-37 | 44 |
| GO:0044283 | small molecule biosynthetic process | 7.76E-36 | 65 |
| GO:1901605 | alpha-amino acid metabolic process | 3.33E-33 | 46 |
| GO:1901607 | alpha-amino acid biosynthetic process | 5.75E-33 | 39 |
| GO:1901564 | organonitrogen compound metabolic process | 1.35E-31 | 148 |
| GO:0009058 | biosynthetic process | 5.20E-29 | 146 |
| GO:1901576 | organic substance biosynthetic process | 1.35E-27 | 143 |
| GO:0044249 | cellular biosynthetic process | 5.51E-26 | 139 |
| GO:0044237 | cellular metabolic process | 2.30E-17 | 188 |
| GO:0006418 | tRNA aminoacylation for protein translation | 2.36E-17 | 19 |
| GO:0071704 | organic substance metabolic process | 6.87E-17 | 185 |
| GO:0008152 | metabolic process | 2.09E-16 | 189 |
| GO:0043038 | amino acid activation | 2.50E-16 | 19 |
| GO:0043039 | tRNA aminoacylation | 2.50E-16 | 19 |
| GO:0009066 | aspartate family amino acid metabolic process | 3.74E-15 | 19 |
| GO:0009067 | aspartate family amino acid biosynthetic process | 2.58E-14 | 16 |
| GO:0046112 | nucleobase biosynthetic process | 7.80E-11 | 11 |
| GO:0009112 | nucleobase metabolic process | 1.04E-09 | 12 |
| GO:0006807 | nitrogen compound metabolic process | 1.05E-09 | 157 |
| GO:0009987 | cellular process | 4.04E-09 | 204 |
| GO:0009069 | serine family amino acid metabolic process | 7.58E-09 | 12 |
| GO:1901137 | carbohydrate derivative biosynthetic process | 9.85E-09 | 28 |
| GO:0090407 | organophosphate biosynthetic process | 1.13E-08 | 28 |
| GO:0055086 | nucleobase-containing small molecule metabolic process | 2.02E-08 | 29 |
| GO:0044238 | primary metabolic process | 2.43E-08 | 159 |
| GO:0006760 | folic acid-containing compound metabolic process | 6.83E-08 | 8 |
| GO:0042558 | pteridine-containing compound metabolic process | 6.83E-08 | 8 |
| GO:0019637 | organophosphate metabolic process | 1.11E-07 | 37 |
| GO:0042398 | cellular modified amino acid biosynthetic process | 1.32E-07 | 10 |
| GO:1901135 | carbohydrate derivative metabolic process | 3.66E-07 | 33 |
| GO:0043094 | cellular metabolic compound salvage | 4.74E-07 | 10 |
| GO:0006575 | cellular modified amino acid metabolic process | 5.39E-07 | 13 |
| GO:0000097 | sulfur amino acid biosynthetic process | 1.05E-06 | 9 |
| GO:0009396 | folic acid-containing compound biosynthetic process | 1.59E-06 | 7 |
| GO:0042559 | pteridine-containing compound biosynthetic process | 1.59E-06 | 7 |
| GO:0009082 | branched-chain amino acid biosynthetic process | 1.78E-06 | 8 |
| GO:0072527 | pyrimidine-containing compound metabolic process | 1.85E-06 | 12 |
| GO:0017144 | drug metabolic process | 1.96E-06 | 25 |
| GO:0098656 | anion transmembrane transport | 4.73E-06 | 13 |
| GO:0051188 | cofactor biosynthetic process | 4.81E-06 | 20 |
| GO:0051186 | cofactor metabolic process | 4.98E-06 | 26 |
| GO:0072528 | pyrimidine-containing compound biosynthetic process | 6.26E-06 | 11 |
| GO:0006730 | one-carbon metabolic process | 9.18E-06 | 6 |
| GO:0043603 | cellular amide metabolic process | 1.10E-05 | 39 |
| GO:0006732 | coenzyme metabolic process | 1.19E-05 | 21 |
| GO:0046942 | carboxylic acid transport | 1.58E-05 | 15 |
| GO:0009081 | branched-chain amino acid metabolic process | 1.62E-05 | 8 |
| GO:0015849 | organic acid transport | 1.93E-05 | 15 |
| GO:0006399 | tRNA metabolic process | 2.04E-05 | 24 |
| GO:0009073 | aromatic amino acid family biosynthetic process | 3.18E-05 | 7 |
| GO:0006553 | lysine metabolic process | 3.56E-05 | 6 |
| GO:0009085 | lysine biosynthetic process | 3.56E-05 | 6 |
| GO:0019856 | pyrimidine nucleobase biosynthetic process | 3.56E-05 | 6 |
| GO:0009070 | serine family amino acid biosynthetic process | 6.69E-05 | 7 |
| GO:0046390 | ribose phosphate biosynthetic process | 8.02E-05 | 12 |
| GO:0000096 | sulfur amino acid metabolic process | 8.54E-05 | 10 |
| GO:0009108 | coenzyme biosynthetic process | 9.95E-05 | 15 |
| GO:0009086 | methionine biosynthetic process | 1.30E-04 | 7 |
| GO:0006820 | anion transport | 1.59E-04 | 21 |
| GO:0043604 | amide biosynthetic process | 1.90E-04 | 34 |
| GO:0009165 | nucleotide biosynthetic process | 2.02E-04 | 14 |
| GO:0006206 | pyrimidine nucleobase metabolic process | 2.52E-04 | 6 |
| GO:1901293 | nucleoside phosphate biosynthetic process | 2.84E-04 | 14 |
| GO:0072522 | purine-containing compound biosynthetic process | 7.07E-04 | 11 |
| GO:0009072 | aromatic amino acid family metabolic process | 7.24E-04 | 8 |
| GO:0006207 | 'de novo' pyrimidine nucleobase biosynthetic process | 7.70E-04 | 5 |
| GO:0009113 | purine nucleobase biosynthetic process | 7.70E-04 | 5 |
| GO:0019878 | lysine biosynthetic process via aminoadipic acid | 7.70E-04 | 5 |
| GO:0055085 | transmembrane transport | 7.73E-04 | 25 |
| GO:0006144 | purine nucleobase metabolic process | 1.04E-03 | 6 |
| GO:0006555 | methionine metabolic process | 1.06E-03 | 7 |
| GO:0034220 | ion transmembrane transport | 1.10E-03 | 15 |
| GO:0015711 | organic anion transport | 1.10E-03 | 18 |
| GO:0015855 | pyrimidine nucleobase transport | 1.11E-03 | 4 |
| GO:0006811 | ion transport | 1.27E-03 | 26 |
| GO:0006566 | threonine metabolic process | 1.99E-03 | 5 |
| GO:0006790 | sulfur compound metabolic process | 2.03E-03 | 16 |
| GO:0006629 | lipid metabolic process | 2.22E-03 | 27 |
| GO:0044272 | sulfur compound biosynthetic process | 2.33E-03 | 11 |
| GO:1903825 | organic acid transmembrane transport | 2.70E-03 | 8 |
| GO:1905039 | carboxylic acid transmembrane transport | 2.70E-03 | 8 |
| GO:0006753 | nucleoside phosphate metabolic process | 3.11E-03 | 19 |
| GO:0009116 | nucleoside metabolic process | 3.55E-03 | 7 |
| GO:0072521 | purine-containing compound metabolic process | 4.30E-03 | 15 |
| GO:0009309 | amine biosynthetic process | 4.36E-03 | 5 |
| GO:0042401 | cellular biogenic amine biosynthetic process | 4.36E-03 | 5 |
| GO:0006015 | 5-phosphoribose 1-diphosphate biosynthetic process | 5.41E-03 | 4 |
| GO:0009095 | aromatic amino acid family biosynthetic process, prephenate pathway | 5.41E-03 | 4 |
| GO:0015851 | nucleobase transport | 5.41E-03 | 4 |
| GO:0046391 | 5-phosphoribose 1-diphosphate metabolic process | 5.41E-03 | 4 |
| GO:0044271 | cellular nitrogen compound biosynthetic process | 5.91E-03 | 69 |
| GO:0006793 | phosphorus metabolic process | 6.14E-03 | 43 |
| GO:0009117 | nucleotide metabolic process | 6.47E-03 | 18 |
| GO:0046148 | pigment biosynthetic process | 7.04E-03 | 7 |
| GO:0009119 | ribonucleoside metabolic process | 8.01E-03 | 6 |
| GO:0000032 | cell wall mannoprotein biosynthetic process | 8.46E-03 | 5 |
| GO:0006056 | mannoprotein metabolic process | 8.46E-03 | 5 |
| GO:0006057 | mannoprotein biosynthetic process | 8.46E-03 | 5 |
| GO:0031506 | cell wall glycoprotein biosynthetic process | 8.46E-03 | 5 |
| GO:0042455 | ribonucleoside biosynthetic process | 8.46E-03 | 5 |
| GO:0006643 | membrane lipid metabolic process | 9.01E-03 | 12 |
| GO:0042440 | pigment metabolic process | 9.64E-03 | 7 |

**Table S4B**

| **GO ID** | **Term name** | **Adjusted p-value** | **Number of genes** |
| --- | --- | --- | --- |
| GO:0044281 | small molecule metabolic process | 1.66E-74 | 139 |
| GO:0043436 | oxoacid metabolic process | 1.62E-51 | 89 |
| GO:0006082 | organic acid metabolic process | 2.13E-51 | 89 |
| GO:0019752 | carboxylic acid metabolic process | 4.82E-50 | 86 |
| GO:0006520 | cellular amino acid metabolic process | 7.70E-40 | 61 |
| GO:1901566 | organonitrogen compound biosynthetic process | 1.86E-31 | 101 |
| GO:0044283 | small molecule biosynthetic process | 9.25E-31 | 61 |
| GO:1901564 | organonitrogen compound metabolic process | 1.72E-27 | 145 |
| GO:1901605 | alpha-amino acid metabolic process | 2.38E-26 | 41 |
| GO:0019637 | organophosphate metabolic process | 2.95E-24 | 60 |
| GO:0016053 | organic acid biosynthetic process | 2.46E-22 | 40 |
| GO:0046394 | carboxylic acid biosynthetic process | 2.46E-22 | 40 |
| GO:0008652 | cellular amino acid biosynthetic process | 1.02E-20 | 32 |
| GO:1901607 | alpha-amino acid biosynthetic process | 3.18E-20 | 30 |
| GO:0009058 | biosynthetic process | 8.36E-20 | 134 |
| GO:1901576 | organic substance biosynthetic process | 3.49E-19 | 132 |
| GO:0009117 | nucleotide metabolic process | 9.73E-19 | 38 |
| GO:0051186 | cofactor metabolic process | 2.79E-18 | 42 |
| GO:0008152 | metabolic process | 4.73E-18 | 197 |
| GO:0006753 | nucleoside phosphate metabolic process | 5.21E-18 | 38 |
| GO:0055086 | nucleobase-containing small molecule metabolic process | 6.75E-18 | 41 |
| GO:0071704 | organic substance metabolic process | 1.55E-17 | 191 |
| GO:0044237 | cellular metabolic process | 1.94E-17 | 193 |
| GO:0044249 | cellular biosynthetic process | 2.79E-16 | 125 |
| GO:1901135 | carbohydrate derivative metabolic process | 3.83E-15 | 45 |
| GO:0006732 | coenzyme metabolic process | 1.49E-14 | 32 |
| GO:0090407 | organophosphate biosynthetic process | 4.11E-13 | 34 |
| GO:0006793 | phosphorus metabolic process | 5.11E-13 | 65 |
| GO:0006796 | phosphate-containing compound metabolic process | 8.24E-13 | 63 |
| GO:0032787 | monocarboxylic acid metabolic process | 3.09E-12 | 29 |
| GO:0017144 | drug metabolic process | 3.43E-12 | 33 |
| GO:1901293 | nucleoside phosphate biosynthetic process | 1.51E-10 | 21 |
| GO:0051188 | cofactor biosynthetic process | 6.92E-10 | 25 |
| GO:0009165 | nucleotide biosynthetic process | 9.24E-10 | 20 |
| GO:1901137 | carbohydrate derivative biosynthetic process | 3.98E-09 | 29 |
| GO:0006733 | oxidoreduction coenzyme metabolic process | 4.43E-09 | 20 |
| GO:0009987 | cellular process | 8.33E-09 | 209 |
| GO:0019693 | ribose phosphate metabolic process | 3.41E-08 | 22 |
| GO:1901615 | organic hydroxy compound metabolic process | 4.95E-08 | 22 |
| GO:0009108 | coenzyme biosynthetic process | 5.11E-08 | 19 |
| GO:0072524 | pyridine-containing compound metabolic process | 5.43E-08 | 18 |
| GO:0006811 | ion transport | 5.81E-08 | 34 |
| GO:0019362 | pyridine nucleotide metabolic process | 6.77E-08 | 17 |
| GO:0009123 | nucleoside monophosphate metabolic process | 1.00E-07 | 19 |
| GO:0044282 | small molecule catabolic process | 1.02E-07 | 20 |
| GO:0009126 | purine nucleoside monophosphate metabolic process | 2.23E-07 | 18 |
| GO:0009167 | purine ribonucleoside monophosphate metabolic process | 2.23E-07 | 18 |
| GO:0009161 | ribonucleoside monophosphate metabolic process | 3.46E-07 | 18 |
| GO:0046496 | nicotinamide nucleotide metabolic process | 3.99E-07 | 16 |
| GO:0006807 | nitrogen compound metabolic process | 4.46E-07 | 154 |
| GO:0006790 | sulfur compound metabolic process | 6.74E-07 | 21 |
| GO:0006629 | lipid metabolic process | 6.94E-07 | 34 |
| GO:0006163 | purine nucleotide metabolic process | 7.61E-07 | 19 |
| GO:0009259 | ribonucleotide metabolic process | 7.61E-07 | 19 |
| GO:0044238 | primary metabolic process | 7.85E-07 | 159 |
| GO:0044255 | cellular lipid metabolic process | 8.22E-07 | 33 |
| GO:0009150 | purine ribonucleotide metabolic process | 2.58E-06 | 18 |
| GO:0009066 | aspartate family amino acid metabolic process | 5.67E-06 | 12 |
| GO:0072521 | purine-containing compound metabolic process | 8.52E-06 | 19 |
| GO:0034220 | ion transmembrane transport | 8.91E-06 | 18 |
| GO:0009067 | aspartate family amino acid biosynthetic process | 1.38E-05 | 10 |
| GO:0043038 | amino acid activation | 1.91E-05 | 11 |
| GO:0043039 | tRNA aminoacylation | 1.91E-05 | 11 |
| GO:0055085 | transmembrane transport | 3.12E-05 | 28 |
| GO:1901617 | organic hydroxy compound biosynthetic process | 4.87E-05 | 14 |
| GO:0009072 | aromatic amino acid family metabolic process | 6.47E-05 | 9 |
| GO:0006418 | tRNA aminoacylation for protein translation | 8.97E-05 | 10 |
| GO:0009124 | nucleoside monophosphate biosynthetic process | 8.97E-05 | 10 |
| GO:0006066 | alcohol metabolic process | 9.72E-05 | 15 |
| GO:0009141 | nucleoside triphosphate metabolic process | 9.72E-05 | 15 |
| GO:0009069 | serine family amino acid metabolic process | 9.80E-05 | 9 |
| GO:0000096 | sulfur amino acid metabolic process | 1.25E-04 | 10 |
| GO:0072528 | pyrimidine-containing compound biosynthetic process | 1.25E-04 | 10 |
| GO:0006090 | pyruvate metabolic process | 1.71E-04 | 10 |
| GO:0046165 | alcohol biosynthetic process | 1.96E-04 | 12 |
| GO:0009127 | purine nucleoside monophosphate biosynthetic process | 3.01E-04 | 9 |
| GO:0009168 | purine ribonucleoside monophosphate biosynthetic process | 3.01E-04 | 9 |
| GO:0000947 | amino acid catabolic process to alcohol via Ehrlich pathway | 3.30E-04 | 6 |
| GO:0000955 | amino acid catabolic process via Ehrlich pathway | 3.30E-04 | 6 |
| GO:0072527 | pyrimidine-containing compound metabolic process | 4.12E-04 | 10 |
| GO:0009132 | nucleoside diphosphate metabolic process | 4.22E-04 | 9 |
| GO:0006576 | cellular biogenic amine metabolic process | 4.63E-04 | 8 |
| GO:0009156 | ribonucleoside monophosphate biosynthetic process | 5.84E-04 | 9 |
| GO:0016054 | organic acid catabolic process | 6.33E-04 | 13 |
| GO:0046395 | carboxylic acid catabolic process | 6.33E-04 | 13 |
| GO:0006812 | cation transport | 9.71E-04 | 18 |
| GO:0046390 | ribose phosphate biosynthetic process | 1.05E-03 | 11 |
| GO:0009199 | ribonucleoside triphosphate metabolic process | 1.06E-03 | 13 |
| GO:0019674 | NAD metabolic process | 1.42E-03 | 8 |
| GO:0009084 | glutamine family amino acid biosynthetic process | 1.43E-03 | 7 |
| GO:0006164 | purine nucleotide biosynthetic process | 1.48E-03 | 10 |
| GO:0009260 | ribonucleotide biosynthetic process | 2.34E-03 | 10 |
| GO:0006113 | fermentation | 2.45E-03 | 6 |
| GO:0098655 | cation transmembrane transport | 2.50E-03 | 12 |
| GO:0055114 | oxidation-reduction process | 2.52E-03 | 19 |
| GO:0044272 | sulfur compound biosynthetic process | 3.44E-03 | 11 |
| GO:0009205 | purine ribonucleoside triphosphate metabolic process | 4.79E-03 | 12 |
| GO:0046034 | ATP metabolic process | 4.79E-03 | 12 |
| GO:0006820 | anion transport | 4.88E-03 | 19 |
| GO:0009308 | amine metabolic process | 5.14E-03 | 9 |
| GO:0044106 | cellular amine metabolic process | 5.14E-03 | 9 |
| GO:0006644 | phospholipid metabolic process | 6.45E-03 | 17 |
| GO:0009144 | purine nucleoside triphosphate metabolic process | 6.52E-03 | 12 |
| GO:0046942 | carboxylic acid transport | 7.58E-03 | 12 |
| GO:0072522 | purine-containing compound biosynthetic process | 7.98E-03 | 10 |
| GO:0009152 | purine ribonucleotide biosynthetic process | 8.09E-03 | 9 |
| GO:0015849 | organic acid transport | 8.78E-03 | 12 |
| GO:0006081 | cellular aldehyde metabolic process | 9.41E-03 | 7 |
