# Supplementary material for: Genome-Wide Analysis of Yeast Metabolic Cycle through Metabolic Network Models Reveals Superiority of Integrated ATAC-seq Data over RNA-seq Data
Source: mSystems. 2022 Jun 13;7(3):e01347-21. doi: 10.1128/msystems.01347-21 (PMC9239220; doi:10.1128/msystems.01347-21)
Supplement: TABLE S5 [file msystems.01347-21-st005.docx]

**Table S5A**

| **KEGG ID** | **Term name** | **Adjusted p-value** | **Number of genes** |
| --- | --- | --- | --- |
| KEGG:01110 | Biosynthesis of secondary metabolites | 3.82E-22 | 47 |
| KEGG:01100 | Metabolic pathways | 5.33E-22 | 62 |
| KEGG:00300 | Lysine biosynthesis | 5.72E-11 | 9 |
| KEGG:01040 | Biosynthesis of unsaturated fatty acids | 3.28E-10 | 8 |
| KEGG:00071 | Fatty acid degradation | 6.56E-10 | 10 |
| KEGG:00620 | Pyruvate metabolism | 1.68E-08 | 13 |
| KEGG:00062 | Fatty acid elongation | 1.47E-07 | 6 |
| KEGG:00380 | Tryptophan metabolism | 3.58E-07 | 8 |
| KEGG:00630 | Glyoxylate and dicarboxylate metabolism | 5.71E-07 | 9 |
| KEGG:01212 | Fatty acid metabolism | 1.04E-06 | 8 |
| KEGG:01200 | Carbon metabolism | 3.68E-06 | 15 |
| KEGG:00410 | beta-Alanine metabolism | 4.65E-06 | 6 |
| KEGG:00310 | Lysine degradation | 1.86E-05 | 6 |
| KEGG:00640 | Propanoate metabolism | 1.01E-04 | 5 |
| KEGG:00010 | Glycolysis / Gluconeogenesis | 1.14E-04 | 9 |
| KEGG:00561 | Glycerolipid metabolism | 1.24E-04 | 7 |
| KEGG:00280 | Valine, leucine and isoleucine degradation | 1.27E-04 | 5 |
| KEGG:01210 | 2-Oxocarboxylic acid metabolism | 2.05E-04 | 7 |
| KEGG:01230 | Biosynthesis of amino acids | 2.19E-04 | 13 |
| KEGG:04146 | Peroxisome | 4.52E-04 | 7 |
| KEGG:00500 | Starch and sucrose metabolism | 5.06E-04 | 7 |
| KEGG:00053 | Ascorbate and aldarate metabolism | 5.81E-04 | 4 |
| KEGG:00230 | Purine metabolism | 6.54E-04 | 8 |
| KEGG:00350 | Tyrosine metabolism | 1.10E-03 | 4 |
| KEGG:00770 | Pantothenate and CoA biosynthesis | 1.17E-03 | 5 |
| KEGG:00340 | Histidine metabolism | 1.39E-03 | 4 |
| KEGG:00040 | Pentose and glucuronate interconversions | 7.83E-03 | 3 |
| KEGG:00330 | Arginine and proline metabolism | 9.23E-03 | 4 |
| KEGG:00592 | alpha-Linolenic acid metabolism | 1.01E-02 | 2 |

**Table S5B**

| **KEGG ID** | **Term name** | **Adjusted p-value** | **Number of genes** |
| --- | --- | --- | --- |
| KEGG:01100 | Metabolic pathways | 3.83E-38 | 97 |
| KEGG:01110 | Biosynthesis of secondary metabolites | 1.63E-24 | 62 |
| KEGG:00010 | Glycolysis / Gluconeogenesis | 1.55E-17 | 24 |
| KEGG:00071 | Fatty acid degradation | 5.42E-14 | 14 |
| KEGG:00620 | Pyruvate metabolism | 2.17E-13 | 20 |
| KEGG:00230 | Purine metabolism | 2.20E-10 | 18 |
| KEGG:00300 | Lysine biosynthesis | 1.44E-09 | 9 |
| KEGG:01240 | Biosynthesis of cofactors | 8.97E-08 | 23 |
| KEGG:01230 | Biosynthesis of amino acids | 1.69E-07 | 22 |
| KEGG:01200 | Carbon metabolism | 5.76E-07 | 20 |
| KEGG:00053 | Ascorbate and aldarate metabolism | 6.80E-07 | 7 |
| KEGG:00350 | Tyrosine metabolism | 2.77E-06 | 7 |
| KEGG:00410 | beta-Alanine metabolism | 2.77E-06 | 7 |
| KEGG:00561 | Glycerolipid metabolism | 4.28E-06 | 10 |
| KEGG:00280 | Valine, leucine and isoleucine degradation | 4.61E-06 | 7 |
| KEGG:00190 | Oxidative phosphorylation | 4.74E-06 | 15 |
| KEGG:00380 | Tryptophan metabolism | 5.41E-06 | 8 |
| KEGG:00310 | Lysine degradation | 1.12E-05 | 7 |
| KEGG:00740 | Riboflavin metabolism | 1.12E-05 | 7 |
| KEGG:01210 | 2-Oxocarboxylic acid metabolism | 6.46E-05 | 9 |
| KEGG:00030 | Pentose phosphate pathway | 7.87E-05 | 8 |
| KEGG:00630 | Glyoxylate and dicarboxylate metabolism | 9.97E-05 | 8 |
| KEGG:00770 | Pantothenate and CoA biosynthesis | 1.51E-04 | 7 |
| KEGG:00640 | Propanoate metabolism | 5.18E-04 | 5 |
| KEGG:00340 | Histidine metabolism | 7.45E-04 | 5 |
| KEGG:00680 | Methane metabolism | 2.41E-03 | 6 |
| KEGG:00730 | Thiamine metabolism | 3.31E-03 | 5 |
| KEGG:00330 | Arginine and proline metabolism | 7.94E-03 | 5 |
| KEGG:01040 | Biosynthesis of unsaturated fatty acids | 2.02E-02 | 3 |
| KEGG:00260 | Glycine, serine and threonine metabolism | 2.41E-02 | 5 |
| KEGG:00592 | alpha-Linolenic acid metabolism | 2.43E-02 | 2 |

**Table S5C**

| **KEGG ID** | **Term name** | **Adjusted p-value** | **Number of genes** |
| --- | --- | --- | --- |
| KEGG:01100 | Metabolic pathways | 9.74E-24 | 64 |
| KEGG:00010 | Glycolysis / Gluconeogenesis | 1.01E-17 | 21 |
| KEGG:00071 | Fatty acid degradation | 1.32E-16 | 14 |
| KEGG:00620 | Pyruvate metabolism | 2.08E-14 | 18 |
| KEGG:01110 | Biosynthesis of secondary metabolites | 1.09E-11 | 36 |
| KEGG:00280 | Valine, leucine and isoleucine degradation | 1.39E-08 | 8 |
| KEGG:00190 | Oxidative phosphorylation | 2.84E-08 | 15 |
| KEGG:01240 | Biosynthesis of cofactors | 3.49E-08 | 19 |
| KEGG:00053 | Ascorbate and aldarate metabolism | 3.76E-08 | 7 |
| KEGG:00410 | beta-Alanine metabolism | 1.67E-07 | 7 |
| KEGG:00350 | Tyrosine metabolism | 5.15E-06 | 6 |
| KEGG:00380 | Tryptophan metabolism | 5.28E-06 | 7 |
| KEGG:00310 | Lysine degradation | 1.75E-05 | 6 |
| KEGG:00740 | Riboflavin metabolism | 1.75E-05 | 6 |
| KEGG:00230 | Purine metabolism | 2.20E-05 | 10 |
| KEGG:00340 | Histidine metabolism | 1.26E-04 | 5 |
| KEGG:00561 | Glycerolipid metabolism | 1.26E-04 | 7 |
| KEGG:00770 | Pantothenate and CoA biosynthesis | 1.44E-04 | 6 |
| KEGG:00330 | Arginine and proline metabolism | 1.45E-03 | 5 |
| KEGG:01200 | Carbon metabolism | 1.45E-03 | 11 |
| KEGG:01040 | Biosynthesis of unsaturated fatty acids | 7.24E-03 | 3 |
| KEGG:00592 | alpha-Linolenic acid metabolism | 1.27E-02 | 2 |
| KEGG:00640 | Propanoate metabolism | 1.47E-02 | 3 |
| KEGG:00630 | Glyoxylate and dicarboxylate metabolism | 2.45E-02 | 4 |
| KEGG:00020 | Citrate cycle (TCA cycle) | 2.97E-02 | 4 |

**Table S5D**

| **KEGG ID** | **Term name** | **Adjusted p-value** | **Number of genes** |
| --- | --- | --- | --- |
| KEGG:00052 | Galactose metabolism | 1.51E-07 | 7 |
| KEGG:01100 | Metabolic pathways | 1.51E-07 | 26 |
| KEGG:00500 | Starch and sucrose metabolism | 1.91E-07 | 8 |
| KEGG:00740 | Riboflavin metabolism | 1.91E-07 | 6 |
| KEGG:00230 | Purine metabolism | 3.38E-05 | 7 |
| KEGG:01240 | Biosynthesis of cofactors | 1.00E-03 | 8 |
| KEGG:00240 | Pyrimidine metabolism | 2.88E-02 | 3 |
