# Supplementary figures and images for: Genome-Wide Analysis of Yeast Metabolic Cycle through Metabolic Network Models Reveals Superiority of Integrated ATAC-seq Data over RNA-seq Data
Source: mSystems. 2022 Jun 13;7(3):e01347-21. doi: 10.1128/msystems.01347-21 (PMC9239220; doi:10.1128/msystems.01347-21)

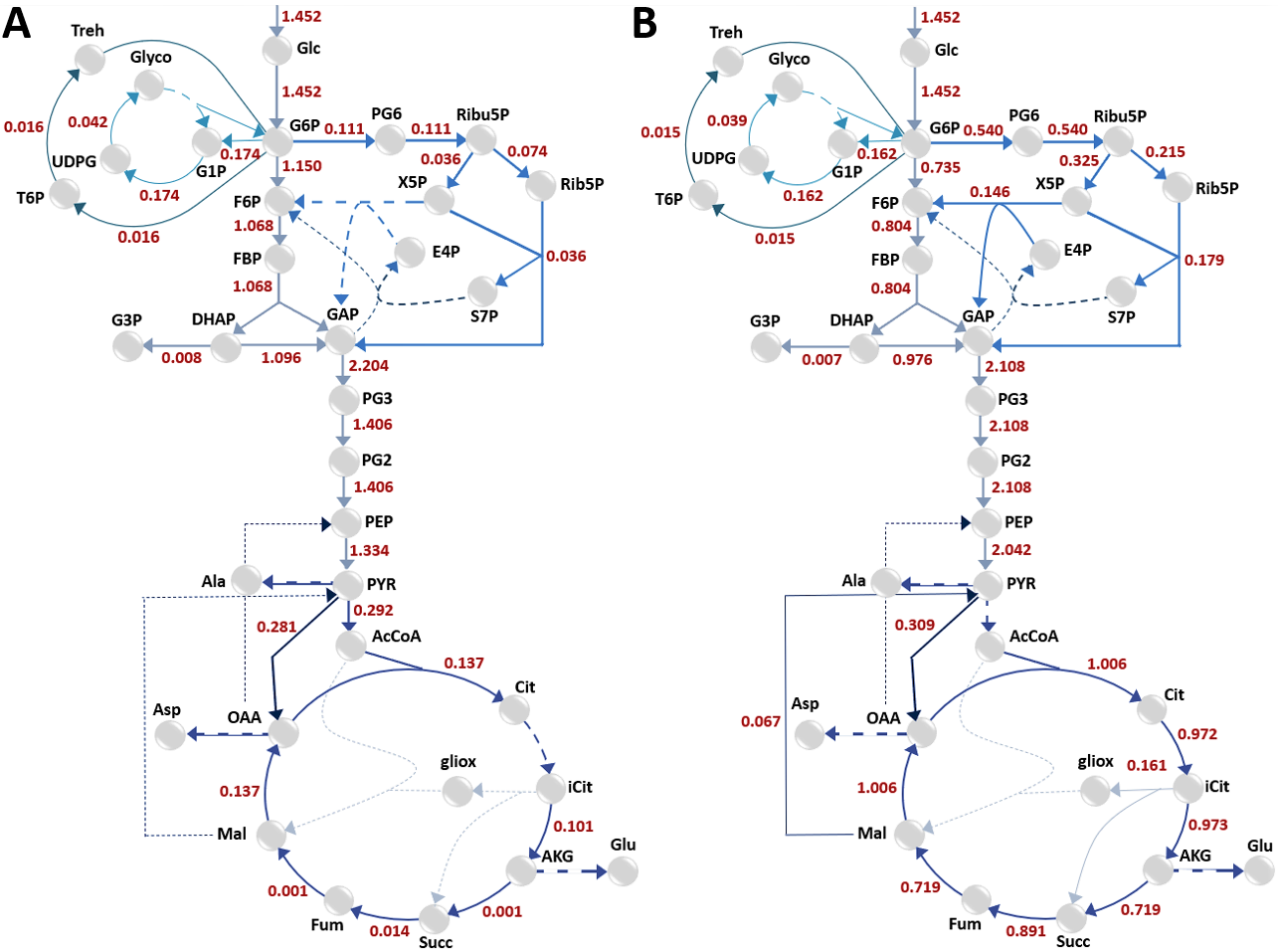

Supplement: FIG S1 [file msystems.01347-21-sf001.tif]

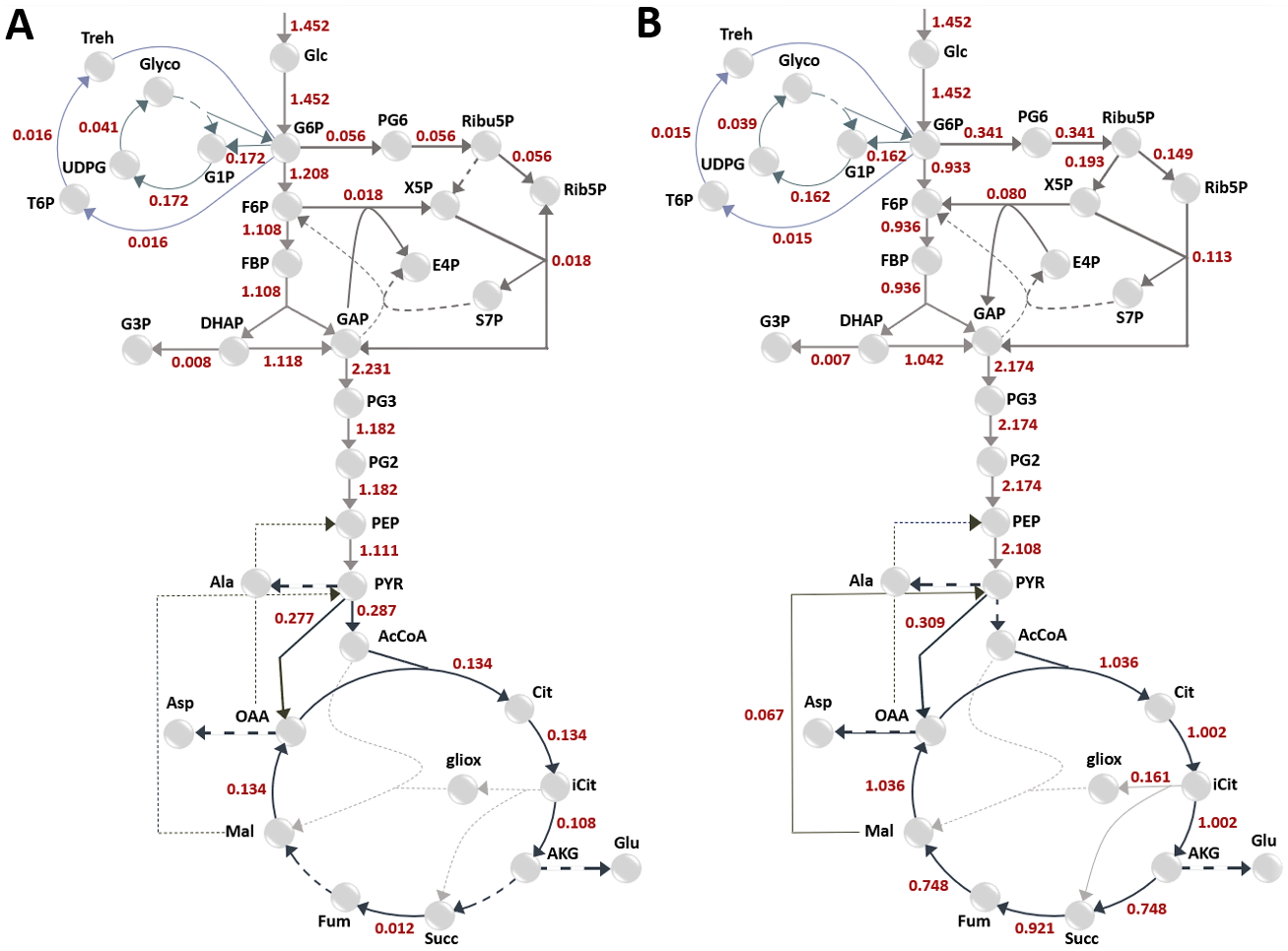

Supplement: FIG S2 [file msystems.01347-21-sf002.tif]

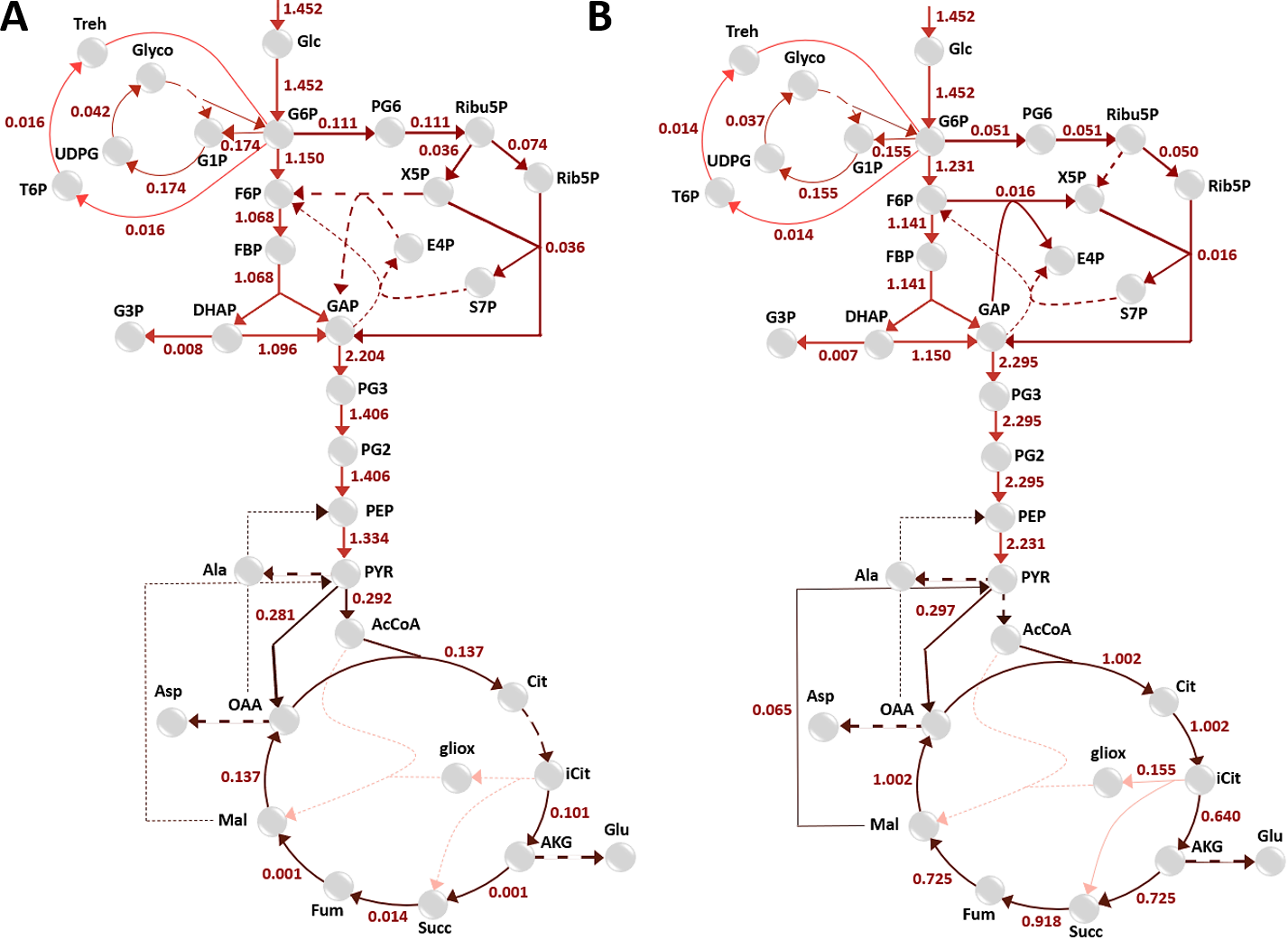

Supplement: FIG S3 [file msystems.01347-21-sf003.tif]
