# Supplementary material for: Genome-Wide Analysis of Yeast Metabolic Cycle through Metabolic Network Models Reveals Superiority of Integrated ATAC-seq Data over RNA-seq Data
Source: mSystems. 2022 Jun 13;7(3):e01347-21. doi: 10.1128/msystems.01347-21 (PMC9239220; doi:10.1128/msystems.01347-21)
Supplement: TABLE S6 [file msystems.01347-21-st006.docx]

**Table S6A**

| **Reaction** | **Reaction Name** | **Early RC (t=87 min)** | **Mid OX (t=10 min)** | **Late RB (t=65 min)** |
| --- | --- | --- | --- | --- |
| r_0534 | hexokinase (D-glucose:ATP) | 1.4493 | 1.3726 | 1.5675 |
| r_4235 | ATP:D-glucose 6-phosphotransferase | 1.4493 | 1.3726 | 1.5675 |
| r_0195 | alpha,alpha-trehalose-phosphate synthase (UDP-forming) | 0.0774 | 0.0395 | 0.0043 |
| r_1051 | trehalose-phosphatase | 0.0773 | 0.0415 | 0.0037 |
| r_0888 | phosphoglucomutase | 0.3974 | 0.2959 | -0.1194 |
| r_1084 | UTP-glucose-1-phosphate uridylyltransferase | 0.4788 | 0.3725 | 0.1711 |
| r_0510 | glycogen (starch) synthase | 0.4001 | 0.3375 | 0.1694 |
| r_0511 | glycogen phosphorylase | 0.0815 | 0.0763 | 0.2908 |
| r_0467 | glucose-6-phosphate isomerase | 0.8648 | 0.9395 | 1.1632 |
| r_0886 | phosphofructokinase | 0.7577 | 0.8541 | 1.414 |
| r_0449 | fructose-bisphosphatase | -0.7577 | -0.8541 | -1.414 |
| r_0450 | fructose-bisphosphate aldolase | 0.7577 | 0.8522 | 1.4158 |
| r_1054 | triose-phosphate isomerase | 0.7583 | 0.8493 | 1.4182 |
| r_0486 | glyceraldehyde-3-phosphate dehydrogenase | 1.4702 | 1.652 | 2.9706 |
| r_0892 | phosphoglycerate kinase | 1.4702 | 1.652 | 2.9706 |
| r_0893 | phosphoglycerate mutase | 1.4035 | 1.5772 | 2.8835 |
| r_0366 | enolase | 1.403 | 1.5786 | 2.8826 |
| r_0962 | pyruvate kinase | 1.4103 | 1.595 | 2.888 |
| r_0961 | pyruvate dehydrogenase | 1.14 | 1.2 | 2.7 |
| r_0491 | glycerol-3-phosphate dehydrogenase (NAD) | -0.0002 | 0.0019 | -0.0015 |
| r_0466 | glucose 6-phosphate dehydrogenase | 0.0196 | 0.0196 | 0.57 |
| r_0091 | 6-phosphogluconolactonase | 0.0196 | 0.0196 | 0.57 |
| r_0889 | phosphogluconate dehydrogenase | 0.0209 | 0.0214 | 0.567 |
| r_0984 | ribulose 5-phosphate 3-epimerase | 0.0318 | 0.0364 | 0.3946 |
| r_1050 | transketolase 2 | -0.0258 | -0.0294 | 0.1565 |
| r_1049 | transketolase 1 | -0.0258 | -0.0294 | 0.1565 |
| r_1048 | transaldolase | -0.0313 | -0.0225 | 0.1486 |
| r_0300 | citrate synthase | 1.0006 | 1.1916 | 2.6639 |
| r_4262 | citrate hydroxymutase | 1.0046 | 1.2007 | 2.6492 |
| r_0658 | isocitrate dehydrogenase (NAD+) | 0.867 | 1.1919 | 2.6126 |
| r_0832 | oxoglutarate dehydrogenase (lipoamide) | 0.7538 | 1.019 | 2.564 |
| r_1021 | succinate dehydrogenase (ubiquinone-6) | 0.8908 | 1.0338 | 2.5958 |
| r_0451 | fumarase | 0.8906 | 1.0257 | 2.6016 |
| r_0713 | malate dehydrogenase | 1.0082 | 0.9765 | 2.6465 |
| r_0662 | isocitrate lyase | 0.1393 | 0.008 | 0.0361 |
| r_0716 | malate synthase | 0.1394 | 0.0084 | 0.0361 |
| r_0470 | glutamate dehydrogenase (NAD) | -0.1157 | -0.1685 | -0.0497 |
| r_0217 | aspartate transaminase | 0.1341 | 0.0328 | 0.0971 |
| r_0674 | L-alanine transaminase | -0.0748 | -0.081 | -0.0042 |
| r_0958 | pyruvate carboxylase | 0.1462 | 0.2676 | 0.1341 |
| r_0884 | phosphoenolpyruvate carboxykinase | 0.0196 | 0.0196 | 0.0196 |
| r_0718 | malic enzyme (NAD) | 0.0196 | 0.0196 | 0.0196 |
| r_0982 | ribose-5-phosphate isomerase | -0.011 | -0.0225 | 0.1486 |

**Table S6B**

| **Reaction** | **Reaction Name** | **Early RC Models** | **Mid OX Models** | **Late RB Models** |
| --- | --- | --- | --- | --- |
| r_0091 | 6-phosphogluconolactonase | 0.0196 | 0.0196 | 0.57 |
| r_0195 | alpha,alpha-trehalose-phosphate synthase (UDP-forming) | 0.0774 | 0.0395 | 0.0043 |
| r_0217 | aspartate transaminase | 0.1341 | 0.0328 | 0.0971 |
| r_0300 | citrate synthase | 1.0006 | 1.1916 | 2.6639 |
| r_0366 | enolase | 1.403 | 1.5786 | 2.8826 |
| r_0449 | fructose-bisphosphatase | -0.7577 | - | -1.414 |
| r_0450 | fructose-bisphosphate aldolase | 0.7577 | 0.8522 | 1.4158 |
| r_0451 | fumarase | 0.8906 | 1.0257 | 2.6016 |
| r_0466 | glucose 6-phosphate dehydrogenase | 0.0196 | 0.0196 | 0.57 |
| r_0467 | glucose-6-phosphate isomerase | 0.8648 | 0.9395 | 1.1632 |
| r_0470 | glutamate dehydrogenase (NAD) | -0.1157 | - | -0.0497 |
| r_0486 | glyceraldehyde-3-phosphate dehydrogenase | 1.4702 | 1.652 | 2.9706 |
| r_0491 | glycerol-3-phosphate dehydrogenase (NAD) | -0.0002 | 0.0019 | -0.0015 |
| r_0510 | glycogen (starch) synthase | 0.4001 | 0.3375 | 0.1694 |
| r_0511 | glycogen phosphorylase | 0.0815 | - | 0.2908 |
| r_0658 | isocitrate dehydrogenase (NAD+) | 0.867 | 1.1919 | 2.6126 |
| r_0662 | isocitrate lyase | 0.1393 | 0.008 | 0.0361 |
| r_0674 | L-alanine transaminase | -0.0748 | -0.081 | -0.0042 |
| r_0713 | malate dehydrogenase | 1.0082 | 0.9765 | 2.6465 |
| r_0716 | malate synthase | 0.1394 | 0.0084 | 0.0361 |
| r_0832 | oxoglutarate dehydrogenase (lipoamide) | 0.7538 | 1.019 | 2.564 |
| r_0886 | phosphofructokinase | 0.7577 | 0.8541 | 1.414 |
| r_0888 | phosphoglucomutase | 0.3974 | 0.2959 | -0.1194 |
| r_0889 | phosphogluconate dehydrogenase | 0.0209 | 0.0214 | 0.567 |
| r_0892 | phosphoglycerate kinase | 1.4702 | 1.652 | 2.9706 |
| r_0893 | phosphoglycerate mutase | 1.4035 | 1.5772 | 2.8835 |
| r_0958 | pyruvate carboxylase | 0.1462 | 0.2676 | 0.1341 |
| r_0962 | pyruvate kinase | 1.4103 | 1.595 | 2.888 |
| r_0984 | ribulose 5-phosphate 3-epimerase | 0.0318 | 0.0364 | - |
| r_1021 | succinate dehydrogenase (ubiquinone-6) | 0.8908 | 1.0338 | 2.5958 |
| r_1048 | transaldolase | -0.0313 | -0.0225 | 0.1486 |
| r_1049 | transketolase 1 | -0.0258 | -0.0294 | 0.1565 |
| r_1050 | transketolase 2 | -0.0258 | -0.0294 | 0.1565 |
| r_1051 | trehalose-phosphatase | 0.0773 | 0.0415 | 0.0037 |
| r_1054 | triose-phosphate isomerase | 0.7583 | 0.8493 | 1.4182 |
| r_1084 | UTP-glucose-1-phosphate uridylyltransferase | 0.4788 | 0.3725 | 0.1711 |
| r_0982 | ribose-5-phosphate isomerase | -0.011 | -0.0225 | 0.1486 |
| r_4235 | ATP:D-glucose 6-phosphotransferase | 1.4493 | 1.3726 | 1.5675 |
| r_0718 | malic enzyme (NAD) | - | 0.0196 | - |
| r_4262 | citrate hydroxymutase | - | 1.2007 | - |
| r_0884 | phosphoenolpyruvate carboxykinase | - | - | 0.0196 |
