# Supplementary material for: Genome-Wide Analysis of Yeast Metabolic Cycle through Metabolic Network Models Reveals Superiority of Integrated ATAC-seq Data over RNA-seq Data
Source: mSystems. 2022 Jun 13;7(3):e01347-21. doi: 10.1128/msystems.01347-21 (PMC9239220; doi:10.1128/msystems.01347-21)

|  |  | **Early RC Phase** | | | | **Mid OX Phase** | | | | | **Late RB Phase** | | | | | | | | |
| --- | --- | --- | --- | --- | --- | --- | --- | --- | --- | --- | --- | --- | --- | --- | --- | --- | --- | --- | --- |
|  | **Thresholds (percentile):** | **25** | **35** | **45** | **50** | **25** | **35** | | **45** | **50** | **25** | **35** | | | | **45** | | **50** | |
| **Mean Squared Error (MSE)** | Yeast8 | 0.182 | 0.196 | 0.196 | 0.185 | 0.239 | 0.315 | | 0.315 | 0.315 | 1.375 | 1.452 | | | | 1.449 | | 1.393 | |
|  | RNA-seq based model | 0.176 | 0.189 | 0.189 | 0.178 | 0.242 | 0.249 | | 0.249 | 0.249 | 1.322 | 1.395 | | | | 1.392 | | 1.313 | |
|  | ATAC-seq based model | 0.113 | 0.142 | 0.142 | 0.130 | 0.066 | 0.136 | | 0.113 | 0.114 | 0.644 | 0.694 | | | | 0.692 | | 0.637 | |
|  | Intersection model | 0.109 | 0.201 | 0.142 | 0.130 | 0.103 | 0.134 | | 0.060 | 0.112 | 0.634 | 1.411 | | | | 0.692 | | 0.636 | |
|  | Union model | 0.182 | 0.196 | 0.196 | 0.185 | 0.243 | 0.318 | | 0.318 | 0.320 | 1.375 | 1.452 | | | | 1.449 | | 1.393 | |
| **Pearson's Correlation Coefficient (r)** | Yeast8 | 0.760 | 0.752 | 0.752 | 0.771 | 0.732 | 0.663 | | 0.663 | 0.663 | 0.586 | 0.575 | | | | 0.577 | | 0.583 | |
|  | RNA-seq based model | 0.774 | 0.767 | 0.767 | 0.786 | 0.731 | 0.721 | | 0.721 | 0.721 | 0.606 | 0.595 | | | | 0.596 | | 0.613 | |
|  | ATAC-seq based model | 0.901 | 0.919 | 0.918 | 0.943 | 0.946 | 0.898 | | 0.911 | 0.910 | 0.849 | 0.847 | | | | 0.844 | | 0.859 | |
|  | Intersection model | 0.914 | 0.764 | 0.918 | 0.943 | 0.875 | 0.884 | | 0.939 | 0.891 | 0.854 | 0.587 | | | | 0.844 | | 0.858 | |
|  | Union model | 0.760 | 0.752 | 0.752 | 0.771 | 0.729 | 0.662 | | 0.662 | 0.660 | 0.586 | 0.575 | | | | 0.577 | | 0.583 | |
|  |  |  |  |  |  |  | |  | | |  | |  |  |  | |  | |  |


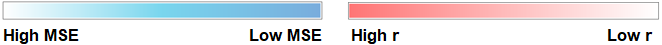

Supplement: TABLE S7 [file msystems.01347-21-st007.docx]
